# Global trends and health workforce analysis of breast cancer burden from high red meat consumption 1990–2050 using machine learning approach

**DOI:** 10.3389/fnut.2025.1576043

**Published:** 2025-08-14

**Authors:** Yuzhou Cai, Jingxian Qian

**Affiliations:** ^1^Department of Gastrointestinal Surgery, The First Affiliated Hospital of Kunming Medical University, Yunnan, China; ^2^Department of Breast Surgery, The First Affiliated Hospital of Kunming Medical University, Yunnan, China

**Keywords:** breast cancer, red meat consumption, Global Burden of Disease, health disparities, healthcare workforce density

## Abstract

**Background:**

High red meat consumption has been implicated in breast cancer development, yet comprehensive global burden assessments and health system relationships remain limited.

**Methods:**

We analyzed breast cancer mortality and disability-adjusted life years (DALYs) using Global Burden of Disease 2021 data across 204 countries. Age-period-cohort analysis, decomposition analysis, health inequality assessment, frontier analysis, and correlation analysis with healthcare workforce density were employed. Machine learning models (ARIMA, Prophet) provided projections to 2050.

**Results:**

Despite declining global age-standardized mortality rates (APC: −0.772%), absolute breast cancer deaths increased from 45,074 (1990) to 81,506 (2021), with DALYs rising from 1.4 to 2.5 million. Profound regional disparities emerged: high-income regions showed declining trends (Western Europe APC: −1.736%) while developing regions experienced increasing burdens (North Africa/Middle East APC: +2.026%). Decomposition analysis revealed population growth (100.266%) and aging (34.86%) as primary drivers, partially offset by epidemiological improvements (−35.127%). Turkey exhibited the largest mortality increase (APC: +3.924%) vs. Denmark's greatest decline (APC: −2.379%). Healthcare workforce analysis demonstrated strong initial correlations between nursing/midwifery density and disease burden (*r* = 0.68, 1990) that weakened substantially over time (*r* = 0.24, 2019), suggesting evolving detection-prevention dynamics. Health inequality analysis showed declining relative disparities (Concentration Index: 0.461–0.297) despite increasing absolute gaps. Machine learning projections forecast continued burden increases, with female deaths reaching 99,749 by 2050.

**Conclusions:**

The global breast cancer burden associated with red meat consumption presents a complex paradox of declining age-standardized rates alongside rising absolute burden, with pronounced inequalities between developed and developing nations. The evolving relationship between healthcare workforce and disease burden highlights shifting dynamics from detection-driven increases to prevention-focused reductions. Strategic policy interventions should prioritize nursing and physical therapy workforce investment in developing regions, implement age-specific prevention strategies for younger populations (25–34 years), and establish context-specific dietary guidelines that consider socioeconomic factors to effectively reduce global breast cancer burden.

## Introduction

Breast cancer remains one of the most significant public health challenges worldwide, accounting for a substantial proportion of cancer-related morbidity and mortality (1). As of 2021, breast cancer burden has increased substantially, with notable disparities in disease outcomes across different regions, genders, and socio-demographic groups (2). Among the various modifiable risk factors, dietary habits, particularly high red meat consumption, have been identified as significant contributors to the burden of breast cancer. Studies have shown a positive association between red meat intake and breast cancer risk, particularly among women, due to factors such as the generation of carcinogenic compounds during high-temperature cooking and the presence of heme iron in red meat (3). Recent meta-analyses have consistently demonstrated that high red meat consumption is associated with increased breast cancer risk, with studies showing a 9–25% increased risk among women with the highest vs. lowest red meat intake (4, 5). Furthermore, the Global Burden of Disease study has identified high red meat intake as accounting for the highest proportion of breast cancer disability-adjusted life years among dietary risk factors (6). These factors underline the importance of understanding the spatiotemporal trends and the influence of socio-demographic factors on breast cancer burden globally.

The global burden of breast cancer has shown an overall increasing trend over the past three decades, driven by factors such as population growth, aging, and changes in epidemiological patterns (7). However, age-standardized mortality rates (ASMR) and disability-adjusted life years (DALYs) have declined in some regions, particularly high-SDI countries, due to advancements in healthcare and early detection programs. By contrast, low- and middle-SDI regions have experienced rising burden trends, often due to limited healthcare resources, late-stage diagnoses, and increasing prevalence of modifiable risk factors such as high red meat consumption (8). Recent studies have specifically highlighted that the burden of breast cancer attributable to high red meat consumption has increased substantially in low- and middle-SDI regions, with deaths rising from 44,492 in 1990 to 79,956 in 2021 globally, while high-SDI regions showed declining age-standardized mortality rates (9, 10). These divergent trends highlight the critical need for tailored interventions and predictive tools to address breast cancer burden globally.

While breast cancer predominantly affects women, the burden among men is also increasing, albeit at a slower rate. Gender-specific differences in incidence, mortality, and DALYs emphasize the need for targeted prevention and treatment strategies (11). Young women aged 25–34 years have shown the most significant increase in DALYs, raising concerns about early exposure to modifiable risk factors, including dietary habits (12, 13). This trend is particularly concerning given that recent evidence suggests the rising global burden of breast cancer in adult women aged 25–45 years associated with high red meat consumption, with pronounced impacts especially in low and middle SDI regions (9). Moreover, the growing burden among younger populations underscores the importance of developing age-specific strategies for breast cancer prevention, particularly in regions with limited healthcare infrastructure.

Predicting the future burden of breast cancer is critical for guiding public health policies and resource allocation. Traditional forecasting methods, while useful, may overlook complex patterns and interactions between risk factors. Machine learning models, such as Prophet (14), ARIMA (15), TBATS (16), Elastic Net (17), ETS (18), VAR (19), Holt-Winters (20), and Theta (21) offer advanced analytical capabilities to capture non-linear relationships and generate more accurate projections. Recent studies have demonstrated the efficacy of these models in predicting disease trends, providing a robust foundation for evidence-based policymaking (22). The application of machine learning in breast cancer burden projections offers a novel and comprehensive approach to addressing global disparities in disease outcomes.

This study aims to provide a comprehensive evaluation of the global breast cancer burden attributable to high red meat consumption, with a focus on spatiotemporal trends, gender- and age-specific differences, and socio-demographic disparities. Using data from the Global Burden of Disease (GBD) study, we assessed the trends in mortality and DALYs from 1990 to 2021 and employed eight machine learning algorithms to project the burden from 2022 to 2050. The results provide critical insights into the dynamics of breast cancer burden and offer a robust evidence base for developing targeted interventions and policies to address modifiable risk factors and reduce health inequities worldwide (23).

## Materials and methods

### Data source

This study utilized two authoritative data sources to analyze and predict the global burden of breast cancer attributable to high red meat consumption. The Global Burden of Disease Study 2021 (GBD 2021) database provided data on age-standardized rates (ASR) for breast cancer mortality and disability-adjusted life years (DALYs) from 1990 to 2021 across 204 countries and territories, encompassing 371 diseases and injuries and 88 risk factors (https://ghdx.healthdata.org/gbd-2021) (24). Population data spanning from 1990 to 2050 were retrieved from published literature (25). The data selected from the GBD database included “Diet high in red meat” as the risk factor and “Breast cancer” as the outcome. Two metrics were analyzed: “Number,” representing absolute counts, and “Rate,” representing the measure per 100,000 population. The analysis focused on individuals aged 25 years and above, based on data availability and the relevance of this demographic to disease burden trends.

### Risk factor definition

High red meat consumption was defined according to the GBD 2021 comparative risk assessment framework. In the GBD methodology, “diet high in red meat” refers to consumption levels exceeding the theoretical minimum risk exposure level (TMREL) of 0–200 g/day. This definition encompasses unprocessed red meat from mammals including beef, pork, lamb, and goat, but excludes processed meat products. The risk estimates are based on systematic reviews and meta-analyses of observational studies that examine the relationship between red meat consumption and breast cancer risk. The GBD framework uses a continuous exposure-response relationship derived from epidemiological evidence to estimate the population attributable fraction for breast cancer cases and deaths attributable to high red meat consumption levels above the TMREL range.

### Disease definition

Breast cancer was defined according to the GBD 2021 framework and international classification systems. It is categorized under the first-level grouping of non-communicable diseases, the second-level grouping of neoplasms, and the third-level grouping of specific cancer types. For standardization and reproducibility, breast cancer was identified using the International Classification of Diseases codes: C50 in ICD-10 and 2C60 in ICD-11 (26, 27).

### Age-period-cohort analysis

Age-period-cohort (APC) analysis was conducted to disentangle the effects of age, time period, and birth cohort on breast cancer mortality and DALYs trends. The analysis employed a Poisson regression model with appropriate constraints to address the inherent identifiability problem in APC models. Age effects were examined across 5-year age groups from 25–29 to 95–99 years. Period effects were analyzed in 5-year intervals from 1990–1994 to 2017–2021. Birth cohort effects were calculated based on the central birth year of each cohort, spanning from 1897–1901 to 1992–1996. Relative risks (RR) with 95% confidence intervals were calculated for each component, with the middle categories serving as reference groups.

### Decomposition analysis

Decomposition analysis was performed to quantify the relative contributions of population growth, population aging, and epidemiological changes to the observed trends in breast cancer burden. The analysis followed the Das Gupta method, decomposing the total change in deaths and DALYs between 1990 and 2021 into three components:

- **Population growth effect**: Changes attributable to increases in population size.
- **Population aging effect**: Changes due to shifts in age structure.
- **Epidemiological effect**: Changes in age-specific rates independent of demographic factors.

The decomposition was stratified by gender, Socio-Demographic Index (SDI) quintiles, and 21 Global Burden of Disease regions to identify differential patterns across demographic and geographic strata.

### Socio-demographic index (SDI) analysis

Countries and territories were categorized into five SDI quintiles (low, low-middle, middle, high-middle, and high) based on income per capita, educational attainment, and total fertility rate. Trend analysis was conducted to examine burden patterns across SDI levels, with annual percentage changes (APCs) calculated using joinpoint regression analysis. The relationship between SDI and disease burden was assessed using Pearson correlation coefficients for both 1990 and 2021.

### Health inequality analysis

Health inequalities were quantified using two complementary measures:

- **Slope Index (SI)**: Measuring absolute inequality by calculating the difference in disease burden between the highest and lowest SDI quintiles
- **Concentration Index (CI)**: Measuring relative inequality by assessing the degree of socioeconomic concentration in disease burden.

Both indices were calculated annually from 1990 to 2021, with 95% confidence intervals estimated using bootstrap methods with 1,000 replications.

### Frontier analysis

Frontier analysis was performed to identify countries with disease burden levels that deviated significantly from expectations based on their socio-demographic development. A stochastic frontier model was fitted using SDI as the primary predictor of expected breast cancer burden. Countries were ranked based on the deviation between observed and frontier-predicted values, with larger positive deviations indicating worse-than-expected performance relative to socio-demographic status.

### Machine learning modeling

To forecast the global breast cancer burden from 2022 to 2050, eight machine learning algorithms were employed in a systematic three-phase framework: data preparation, model construction, and model evaluation.

During the data preparation phase, extensive preprocessing and feature engineering were conducted to enhance predictive performance. Time-series data from 1990 to 2021 and population data from 1990 to 2050 were standardized for unit consistency to eliminate dimensional inconsistencies. Specifically, this involved: (1) unit standardization—converting all mortality and DALY rates to consistent units (per 100,000 population) and ensuring temporal consistency in reporting periods; (2) scale alignment—adjusting population figures to consistent demographic units (total population counts) to match the rate calculations; and (3) temporal alignment—ensuring all time-series data points correspond to the same calendar years and age groups for accurate trend analysis. No statistical normalization techniques (such as *z*-score standardization or min-max scaling) were applied to preserve the original scale and clinical interpretability of the epidemiological measures.

In the model construction phase, eight advanced time-series and machine learning models were applied: Prophet, ARIMA, TBATS, Elastic Net, ETS, VAR, Holt-Winters, and Theta. Each model was selected based on its specific strengths and underlying assumptions (Supplementary Table S15). Prophet, a modern time-series forecasting model, was chosen for its ability to automatically detect changepoints and handle missing values through its logistic growth framework. ARIMA (Autoregressive Integrated Moving Average), a classical statistical model, was selected for its solid theoretical foundation and effectiveness in modeling stationary time-series data. TBATS (Trigonometric seasonality, Box-Cox transformation, ARMA errors, Trend and Seasonal components) was included for its capability to handle complex seasonal patterns and multiple seasonalities. Elastic Net, a regularization technique, was chosen to address multicollinearity among predictors while performing automatic feature selection. ETS (Error-Trend-Seasonal) was selected for its simplicity and efficiency in business forecasting applications. VAR (Vector Autoregression) was included to capture dynamic relationships between multiple variables. Holt-Winters exponential smoothing was chosen for its proven effectiveness in handling time-series with trend and seasonality. Finally, Theta method was selected for its simplicity and stable performance in forecasting applications. Each model was carefully tuned to optimize performance. For example, the Prophet model, designed for strong seasonal patterns, utilized a logistic growth framework with changepoints, while the ARIMA model was optimized using automated parameter selection and external covariates, such as population data. Similarly, TBATS captured complex seasonality, Elastic Net addressed multicollinearity among predictors, and VAR modeled dynamic relationships between variables. Holt-Winters and Theta models, known for their robust performance in time-series forecasting, were also enhanced by introducing additive components and constrained periodic growth rates. All models were evaluated using cross-validation to ensure generalizability and accuracy.

The final model evaluation phase involved rigorous performance assessment using multiple metrics to comprehensively evaluate predictive accuracy. These metrics included mean squared error (MSE), mean absolute percentage error (MAPE), root mean squared error (RMSE), symmetric mean absolute percentage error (SMAPE), R-squared (*R*^2^), and mean absolute scaled error (MASE). MSE measures the average squared differences between predicted and actual values, with lower values indicating better model performance. MAPE represents the mean absolute percentage difference between predicted and actual values, providing a scale-independent measure of prediction accuracy. RMSE is the square root of MSE, offering interpretability in the same units as the original data. SMAPE addresses the asymmetry issues in MAPE by using the average of predicted and actual values in the denominator. *R*^2^ indicates the proportion of variance in the dependent variable explained by the model, with values closer to 1 indicating better model fit. MASE compares the forecast accuracy against a naive seasonal forecast, with values <1 indicating better performance than the baseline model. Cross-validation was implemented using a time-series approach, where data from 1990 to 2010 served as the training set, 2011 to 2021 as the validation set, and 2022 to 2050 as the test set. This approach ensured the robustness of both short-term and long-term predictions.

### Human resources for health data

Data on human resources for health (HRH) were retrieved from the GBD 2019 Health Workforce Collaborators, providing density metrics (workers per 10,000 population) for 22 distinct workforce categories across 204 countries and territories for 1990 and 2019. These categories encompassed the full spectrum of healthcare providers, from physicians and nursing professionals to allied health workers and traditional practitioners, enabling comprehensive analysis of associations between healthcare workforce density and breast cancer burden metrics.

### Statistical analysis

In addition to machine learning predictions, several advanced statistical methods were employed to interpret trends in breast cancer burden. Estimated annual percentage change (EAPC) was calculated using a linear regression model fitted to the natural logarithm of ASRs over time, providing insights into temporal trends. An age-period-cohort (APC) model was constructed using a generalized linear framework to disentangle the effects of age, period, and cohort on breast cancer burden. Decomposition analysis quantified the contributions of population growth, aging, and epidemiological changes to variations in burden, while socio-demographic index (SDI) analysis explored the relationship between SDI and disease burden using LOESS smoothing and Spearman's rank correlation. Frontier analysis was conducted using data envelopment analysis (DEA) to assess countries' relative performance in health improvement. Health inequality was analyzed using the Slope Index of Inequality (SII) and the Concentration Index (CI) to evaluate absolute and relative disparities, respectively.

## Results

### Global burden of breast cancer attributable to high red meat consumption among adults aged 25 and above, 1990–2021

**Figure 1:**
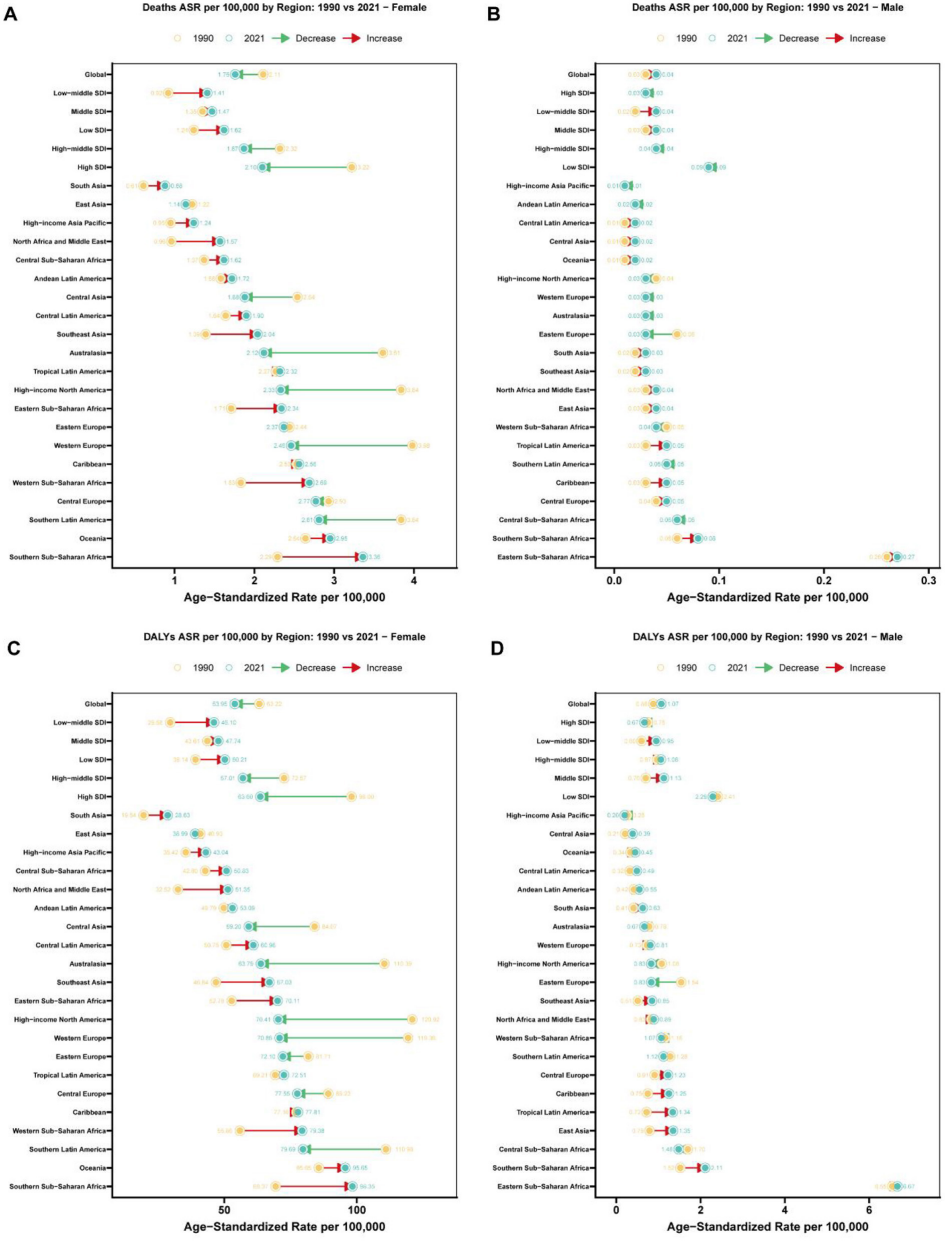
Regional comparison of age-standardized rates for breast cancer attributable to high red meat diet consumption: 1990 vs. 2021. **(A)** Age-standardized death rates (per 100,000 population) for breast cancer attributable to high red meat diet consumption among females across 27 regions (Global, 5 SDI regions, and 21 GBD regions) in 1990 and 2021. Connected lines with arrows indicate the direction and magnitude of change from 1990 to 2021, with red arrows representing increases and green arrows representing decreases. Regions are dynamically ordered by 2021 age-standardized rates, with Global shown first, followed by SDI regions and then 21 specific regions in ascending order of 2021 rates. Yellow circles represent 1990 rates, and teal circles represent 2021 rates, with exact values labeled. **(B)** Age-standardized death rates (per 100,000 population) for breast cancer attributable to high red meat diet consumption among males across 27 regions (Global, 5 SDI regions, and 21 GBD regions) in 1990 and 2021. Connected lines with arrows indicate the direction and magnitude of change from 1990 to 2021, with red arrows representing increases and green arrows representing decreases. Regions are dynamically ordered by 2021 age-standardized rates, with Global shown first, followed by SDI regions and then 21 specific regions in ascending order of 2021 rates. Yellow circles represent 1990 rates, and teal circles represent 2021 rates, with exact values labeled. **(C)** Age-standardized disability-adjusted life years (DALYs) rates (per 100,000 population) for breast cancer attributable to high red meat diet consumption among females across 27 regions (Global, 5 SDI regions, and 21 GBD regions) in 1990 and 2021. Connected lines with arrows indicate the direction and magnitude of change from 1990 to 2021, with red arrows representing increases and green arrows representing decreases. Regions are dynamically ordered by 2021 age-standardized rates, with Global shown first, followed by SDI regions and then 21 specific regions in ascending order of 2021 rates. Yellow circles represent 1990 rates, and teal circles represent 2021 rates, with exact values labeled. **(D)** Age-standardized disability-adjusted life years (DALYs) rates (per 100,000 population) for breast cancer attributable to high red meat diet consumption among males across 27 regions (Global, 5 SDI regions, and 21 GBD regions) in 1990 and 2021. Connected lines with arrows indicate the direction and magnitude of change from 1990 to 2021, with red arrows representing increases and green arrows representing decreases. Regions are dynamically ordered by 2021 age-standardized rates, with Global shown first, followed by SDI regions and then 21 specific regions in ascending order of 2021 rates. Yellow circles represent 1990 rates, and teal circles represent 2021 rates, with exact values labeled.

The global burden of breast cancer among individuals aged 25 years and above showed a significant upward trend between 1990 and 2021 (Figure 1). As detailed in Supplementary Table S1, breast cancer-related deaths in this population increased from 45,073.853 in 1990 to 81,506.227 in 2021. Age-standardized mortality rates (ASMR) decreased from 1.169 per 100,000 population (95% UI: 0 to 2.498) in 1990 to 0.956 per 100,000 (95% UI: 0 to 2.058) in 2021, with an annual percentage change (APC) of −0.772% (95% confidence interval [CI]: −0.819% to −0.726%).

Gender differences in mortality trends were also observed. Breast cancer-related deaths among males rose from 581.902 in 1990 to 1,549.268 in 2021, accompanied by a slight increase in ASMR, with an APC of 0.628% (95% CI: 0.529% to 0.727%). Conversely, among females, deaths increased from 44,491.951 in 1990 to 79,956.959 in 2021, while ASMR decreased, with an APC of −0.73% (95% CI: −0.779% to −0.682%).

Similarly, as shown in Supplementary Table S2, disability-adjusted life years (DALYs) due to breast cancer among individuals aged 25 years and above increased from 1,396,840.461 in 1990 to 2,451,718.64 (95% UI: −790.88 to 5,232,217.293) in 2021. The age-standardized DALY rate decreased from 33.313 per 100,000 (95% UI: −0.01 to 71.677) in 1990 to 28.367 per 100,000 (95% UI: −0.009 to 60.536) in 2021, with an APC of −0.649% (95% CI: −0.697% to −0.601%). Gender-specific trends showed that male DALYs rose from 17,119.079 in 1990 to 44,626.38 in 2021, with an APC of 0.844% (95% CI: 0.737%−0.951%). In contrast, female DALYs increased from 1,379,721.382 in 1990 to 2,407,092.26 in 2021, while the age-standardized rate declined, with an APC of −0.653% (95% CI: −0.705% to −0.602%).

### Socio-demographic index stratification of breast cancer mortality and DALYs attributable to high red meat intake: global trends, 1990–2021

The burden of breast cancer varied substantially across different Socio-Demographic Index (SDI) quintiles (Figure 1). From 1990 to 2021, ASMR decreased significantly in high-SDI regions, dropping from 1.834 per 100,000 to 1.141 per 100,000, with an APC of −1.626 (95% CI: −1.67 to −1.581) (Supplementary Table S1). In contrast, low-middle SDI regions experienced an increase in ASMR, with an APC of 1.55 (95% CI: 1.519–1.581). For DALYs, high-SDI regions also demonstrated a marked decline, with age-standardized rates dropping from 52.817 per 100,000 in 1990 to 33.073 in 2021, yielding an APC of −1.594 (95% CI: −1.632 to −1.556) (Supplementary Table S2). In contrast, middle-SDI and low-middle SDI regions saw increasing DALY rates, with APCs of 0.262 (95% CI: 0.208–0.316) and 1.558 (95% CI: 1.526–1.591), respectively.

### Regional variation in breast cancer burden attributable to high red meat consumption: a comprehensive analysis of 21 GBD regions

Regional disparities in the burden of breast cancer were evident from 1990 to 2021 (Figure 1). High-income regions such as Western Europe and North America showed significant reductions in ASMR, with APCs of −1.736 (95% CI: −1.785 to −1.686) and −1.855 (95% CI: −1.913 to −1.797), respectively (Supplementary Table S1). In contrast, regions such as North Africa and the Middle East exhibited increasing ASMR, with an APC of 2.026 (95% CI: 1.804–2.248). Similarly, for DALYs, age-standardized rates declined substantially in high-income North America, from 65.486 per 100,000 in 1990 to 37.348 in 2021, with an APC of −1.938 (95% CI: −2.000 to −1.875) (Supplementary Table S2). However, South Asia and Southeast Asia experienced rising DALY rates, with APCs of 1.236 (95% CI: 1.096–1.376) and 1.119 (95% CI: 1.062–1.175), respectively.

### Age-specific patterns of breast cancer mortality and DALYs attributable to high red meat intake: a three-decade global analysis

**Figure 2:**
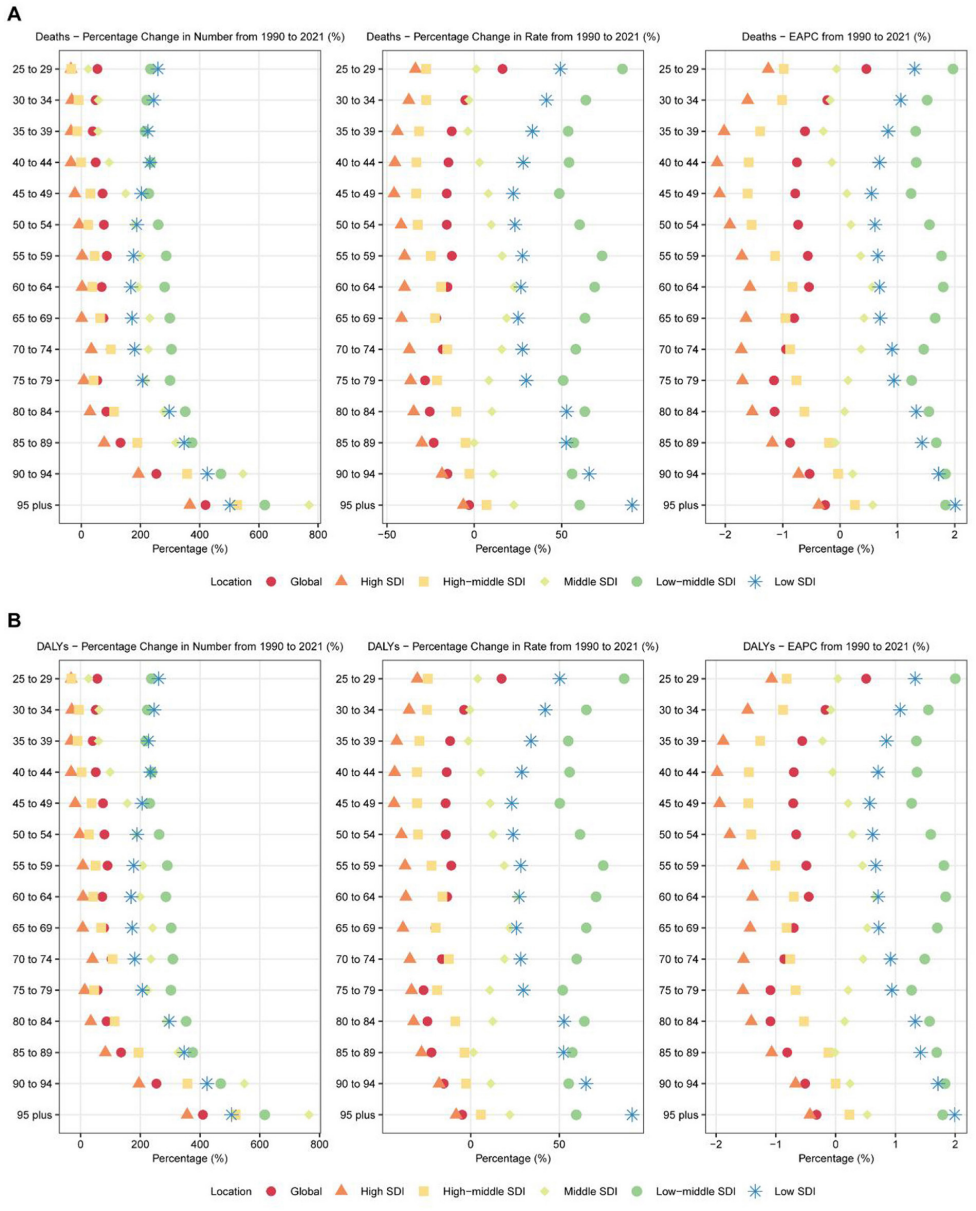
Age-specific trends in breast cancer attributable to high red meat diet consumption across 6 major regions: 1990–2021. **(A)** Age-specific percentage changes in deaths from breast cancer attributable to high red meat diet consumption for both sexes combined across 6 major regions (Global and 5 SDI regions) from 1990 to 2021. The figure displays three metrics: percentage change in absolute numbers (left panel), percentage change in age-specific rates (middle panel), and estimated annual percentage change (EAPC) in age-specific rates (right panel). Each point represents a specific age group (25–29 years to 95+ years) and region, with different colors and shapes distinguishing the six regions. Age groups are arranged from oldest (top) to youngest (bottom). **(B)** Age-specific percentage changes in disability-adjusted life years (DALYs) from breast cancer attributable to high red meat diet consumption for both sexes combined across 6 major regions (Global and 5 SDI regions) from 1990 to 2021. The figure displays three metrics: percentage change in absolute numbers (left panel), percentage change in age-specific rates (middle panel), and estimated annual percentage change (EAPC) in age-specific rates (right panel). Each point represents a specific age group (25–29 years to 95+ years) and region, with different colors and shapes distinguishing the six regions. Age groups are arranged from oldest (top) to youngest (bottom).

The burden of breast cancer demonstrated distinct patterns across age groups between 1990 and 2021 (Figure 2). Among women aged 25 years and above, overall mortality declined, but notable differences emerged between age groups. The most significant reduction in mortality occurred in the 40–44 age group, with an APC of −0.815 (95% CI: −0.939 to −0.692) (Supplementary Table S3). Older age groups, such as 75–79 years, also showed marked declines, with an APC of −0.931 (95% CI: −1.015 to −0.847). However, mortality rates increased in the younger 25–29 age group, with an APC of 0.449 (95% CI: 0.347–0.550), warranting further investigation.

In terms of DALYs, most age groups exhibited declines, though the extent varied. The 50–54 age group experienced the largest decrease in DALYs, with an APC of −0.750 (95% CI: −0.821 to −0.678) (Supplementary Table S4). Older groups, such as 75–79 years and 80–84 years, also showed significant declines, with APCs of −0.868 (95% CI: −0.950 to −0.785) and −0.846 (95% CI: −0.911 to −0.781), respectively. However, DALYs increased in younger age groups, particularly among women aged 25–34 years. The 30–34 age group showed a notable increase, with an APC of 1.479 (95% CI: 1.371–1.587).

For males, while breast cancer remains relatively rare, certain age groups showed increasing burdens. For example, men aged 60–64 years exhibited an APC of 0.832 (95% CI: 0.685–0.980) for mortality and 0.947 (95% CI: 0.791–1.104) for DALYs, highlighting the need to address male breast cancer prevention and treatment.

### Country-level analysis of breast cancer burden attributable to high red meat consumption: disparities across 204 countries and territories

Country-level analysis revealed pronounced heterogeneity in red meat-associated breast cancer mortality and disability burden (Supplementary Tables S5, S6). Among 204 countries, 76 (37.3%) experienced a decrease in age-standardized mortality rates, while 128 (62.7%) showed increasing trends from 1990 to 2021. The most substantial declines in mortality were observed in Denmark (APC: −2.379, 95% CI: −2.475 to −2.282), Greenland (APC: −2.493, 95% CI: −2.653 to −2.332), and the United Kingdom (APC: −2.294, 95% CI: −2.379 to −2.209). Conversely, the most pronounced increases occurred in Turkey (APC: 3.924, 95% CI: 3.186–4.667), Egypt (APC: 2.961, 95% CI: 2.530–3.394), and Zimbabwe (APC: 2.959, 95% CI: 2.278–3.644).

**Figure 3:**
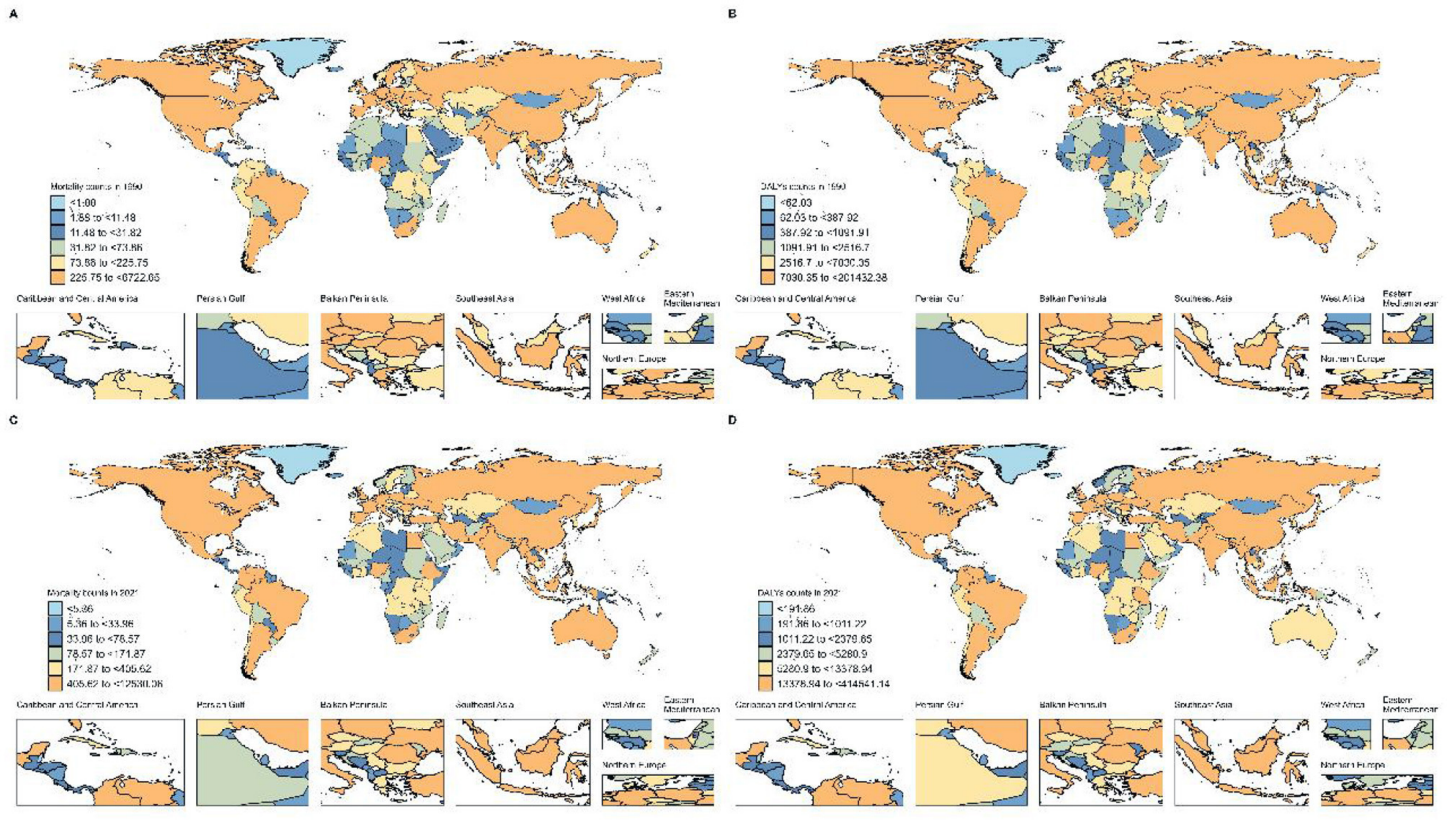
Global Distribution of Breast Cancer Burden Attributable to High Red Meat Consumption. **(A)** Number of deaths in 204 countries, 1990. **(B)** Number of DALYs in 204 countries, 1990. **(C)** Number of deaths in 204 countries, 2021. **(D)** Number of DALYs in 204 countries, 2021.

Similar patterns were observed in disability-adjusted life years attributed to red meat-associated breast cancer, with Denmark showing the most significant improvement (APC: −2.765, 95% CI: −2.878 to −2.651), followed by United Kingdom (APC: −2.390, 95% CI: −2.456 to −2.325) and Norway (APC: −2.338, 95% CI: −2.518 to −2.158). However, Zimbabwe (APC: 3.326, 95% CI: 2.546–4.111), Turkey (APC: 3.779, 95% CI: 3.051–4.513), and Lesotho (APC: 3.035, 95% CI: 2.593–3.478) demonstrated the most dramatic increases in DALY burden. In absolute terms, age-standardized mortality rates for red meat-associated breast cancer in 2021 were highest in South Africa (1.951 per 100,000), Angola (1.397 per 100,000), and Georgia (2.222 per 100,000), while the lowest rates were observed in Bangladesh (0.232 per 100,000), Mongolia (0.446 per 100,000), and Sri Lanka (0.313 per 100,000). For DALYs, the highest age-standardized rates in 2021 were found in Nauru (86.552 per 100,000), American Samoa (82.734 per 100,000), and Fiji (81.052 per 100,000), illustrating the disproportionate burden among Pacific Island nations (Figure 3).

### Gender-specific trends in breast cancer burden attributable to high red meat consumption: a comparative analysis of male and female populations

Significant differences in breast cancer burden were observed among nations. For mortality (Supplementary Table S5), Turkey showed the largest increase, with an APC of 3.924 (95% CI: 3.186–4.667), followed by Malawi (APC: 3.060, 95% CI: 2.961–3.159) and Lesotho (APC: 2.970, 95% CI: 2.554–3.387). Conversely, countries such as Denmark, the United Kingdom, and Malta exhibited significant declines, with APCs of −2.379 (95% CI: −2.475 to −2.282), −2.294 (95% CI: −2.379 to −2.209), and −2.208 (95% CI: −2.358 to −2.058), respectively. Greenland showed the steepest decline, with an APC of −2.493 (95% CI: −2.653 to −2.332). In terms of DALYs (Supplementary Table S6), Turkey also exhibited the highest increase, with an APC of 3.779 (95% CI: 3.051–4.513). Meanwhile, nations such as Denmark, the United Kingdom, and Norway demonstrated significant reductions in DALYs, with APCs of −2.765 (95% CI: −2.878 to −2.651), −2.390 (95% CI: −2.456 to −2.325), and −2.338 (95% CI: −2.518 to −2.158), respectively. These findings underscore the global inequality in breast cancer burden, with developing nations, particularly in Africa, experiencing rapid increases in disease burden, while developed nations have achieved notable progress in reducing breast cancer mortality and DALYs.

### Age-period-cohort analysis of breast cancer burden attributable to high red meat consumption: decomposing temporal effects, 1990–2021

**Figure 4:**
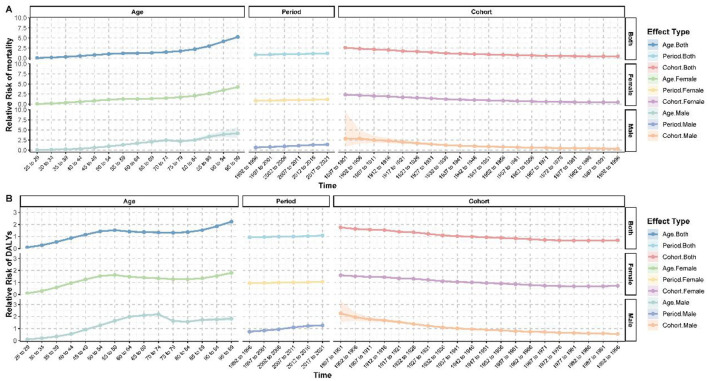
Age-period-cohort analysis of breast cancer burden attributable to high red meat consumption. **(A)** Age-period-cohort effects on mortality. **(B)** Age-period-cohort effects on DALYs.

An age-period-cohort (APC) analysis revealed complex patterns in breast cancer mortality and DALYs (Figure 4). Overall, the risk of breast cancer increased significantly with advancing age (Supplementary Table S7). Compared to the 25–29 age group, the relative risk (RR) of mortality reached 5.258 (95% CI: 5.112–5.409, *p* < 0.001) and the RR of DALYs was 2.235 (95% CI: 2.214–2.256, *p* < 0.001) for individuals aged 95–99 years. Gender differences were also significant, with male breast cancer risks increasing more steeply with age. For instance, the RR of mortality for males aged 90–94 years was 3.876 (95% CI: 3.191–4.708, *p* < 0.001), compared to 3.464 (95% CI: 3.399–3.531, *p* < 0.001) in females of the same age group (Supplementary Table S8).

Period effects showed an overall increase in the breast cancer burden in recent years. During the 2017–2021 period, the RR of mortality was 1.198 (95% CI: 1.189–1.207, *p* < 0.001) and the RR of DALYs was 1.083 (95% CI: 1.081–1.084, *p* < 0.001), compared to the baseline period of 1990–1991. Conversely, cohort effects showed a clear downward trend. Compared to the 1942–1946 birth cohort, the RR of mortality in the 1992–1996 cohort dropped to 0.462 (95% CI: 0.420–0.508, *p* < 0.001), and the RR of DALYs declined to 0.693 (95% CI: 0.685–0.702, *p* < 0.001). This trend was observed in both males and females, though the decline was more pronounced in males (Supplementary Table S9).

Interestingly, age-specific risk patterns varied significantly by gender. For women, the RR of mortality surpassed 1 in the 50–54 age group (RR = 1.119, 95% CI: 1.105–1.133, *p* < 0.001), whereas in men, the RR of mortality only exceeded 1 in the 55–59 age group (RR = 1.313, 95% CI: 1.188–1.452, *p* < 0.001). These findings highlight both age- and gender-specific dynamics in breast cancer risk and burden.

### Decomposition analysis of breast cancer burden attributable to high red meat consumption: population growth, aging, and epidemiological changes

Globally, the burden of breast cancer has grown significantly between 1990 and 2021, though the patterns of change were complex. Over this period, mortality showed an overall increase of 83.838%, while DALYs rose by 3136.062% (Supplementary Tables S10, S11). These changes were primarily driven by population growth (mortality: 100.266%, DALYs: 105.438%) and aging (mortality: 34.86%, DALYs: 23.844%). However, epidemiological changes contributed negatively to these trends (mortality: −35.127%, DALYs: −29.282%), partially offsetting the effects of demographic factors.

Gender differences in these trends were striking. For females, the total change in mortality (83.39%) and DALYs (3121.955%) was far greater than for males (mortality: 0.448%, DALYs: 14.107%), reflecting the predominance of breast cancer in women. Notably, for males, epidemiological changes showed a positive contribution (mortality: 16.363%, DALYs: 21.621%), potentially indicating changes in risk factors or improvements in male breast cancer detection.

Variations in breast cancer burden across SDI regions were also apparent (Supplementary Tables S10, S11). High-SDI regions demonstrated the largest overall change (mortality: 36,432.373%, DALYs: 1,054,878.179%), driven by population growth (mortality: 204.836%, DALYs: 506.675%) and aging (mortality: 164.581%, DALYs: 252.652%). However, negative contributions from epidemiological changes (mortality: −269.417%, DALYs: −659.328%) significantly mitigated these increases. In contrast, low-SDI regions exhibited smaller overall changes (mortality: 147.947%, DALYs: 2,510.288%), primarily driven by population growth (mortality: 78.437%, DALYs: 77.672%), with aging playing a minor or negative role (mortality: −2.533%, DALYs: −1.791%). Middle-SDI regions showed a more balanced contribution pattern, with population growth (mortality: 64.317%, DALYs: 68.965%), aging (mortality: 24.982%, DALYs: 18.252%), and epidemiological changes (mortality: 10.701%, DALYs: 12.783%) all contributing positively. Low-middle SDI regions displayed a unique pattern, with epidemiological changes making a significant positive contribution (mortality: 36.617%, DALYs: 38.027%), likely reflecting increased breast cancer risk factors or improved diagnostic capacity in these regions (Supplementary Figure S1).

Among the 21 Global Burden of Disease (GBD) regions, variation in change patterns was even more pronounced (Supplementary Tables S10, S11). Western Europe experienced the largest overall change in mortality (3,237.326%), driven primarily by aging (1,354.249%) and population growth (1,146.892%), but was significantly offset by negative contributions from epidemiological changes (−2,401.142%). In terms of DALYs, the largest change was observed in Western Sub-Saharan Africa (437,195.83%), driven by population growth (69.828%) and epidemiological changes (33.473%). Conversely, some regions, such as Central Europe, exhibited negative overall changes in DALYs (−39,441.415%), with positive contributions from aging (100.1%) and population growth (68.237%) offset by large negative epidemiological changes (−68.337%).

East Asia demonstrated notable progress in controlling breast cancer, with relatively smaller negative contributions from epidemiological changes (mortality: −4.557%, DALYs: −1.449%) despite significant overall increases in burden (mortality: 2,174.524%, DALYs: 64,809.332%). High-income Asia Pacific regions exhibited a unique trend in DALYs, with overall change reaching 106,872.673%, driven significantly by epidemiological changes (33.651%), highlighting emerging challenges in these areas. Similarly, South and Southeast Asia demonstrated substantial increases in DALYs, with significant contributions from epidemiological changes (South Asia: 34.785%, Southeast Asia: 28.632%), potentially reflecting lifestyle changes and increased breast cancer risk factors in these regions (Supplementary Figure S1).

### Health inequality trends in breast cancer burden attributable to high red meat consumption: global slope index and concentration index analysis

**Figure 5:**
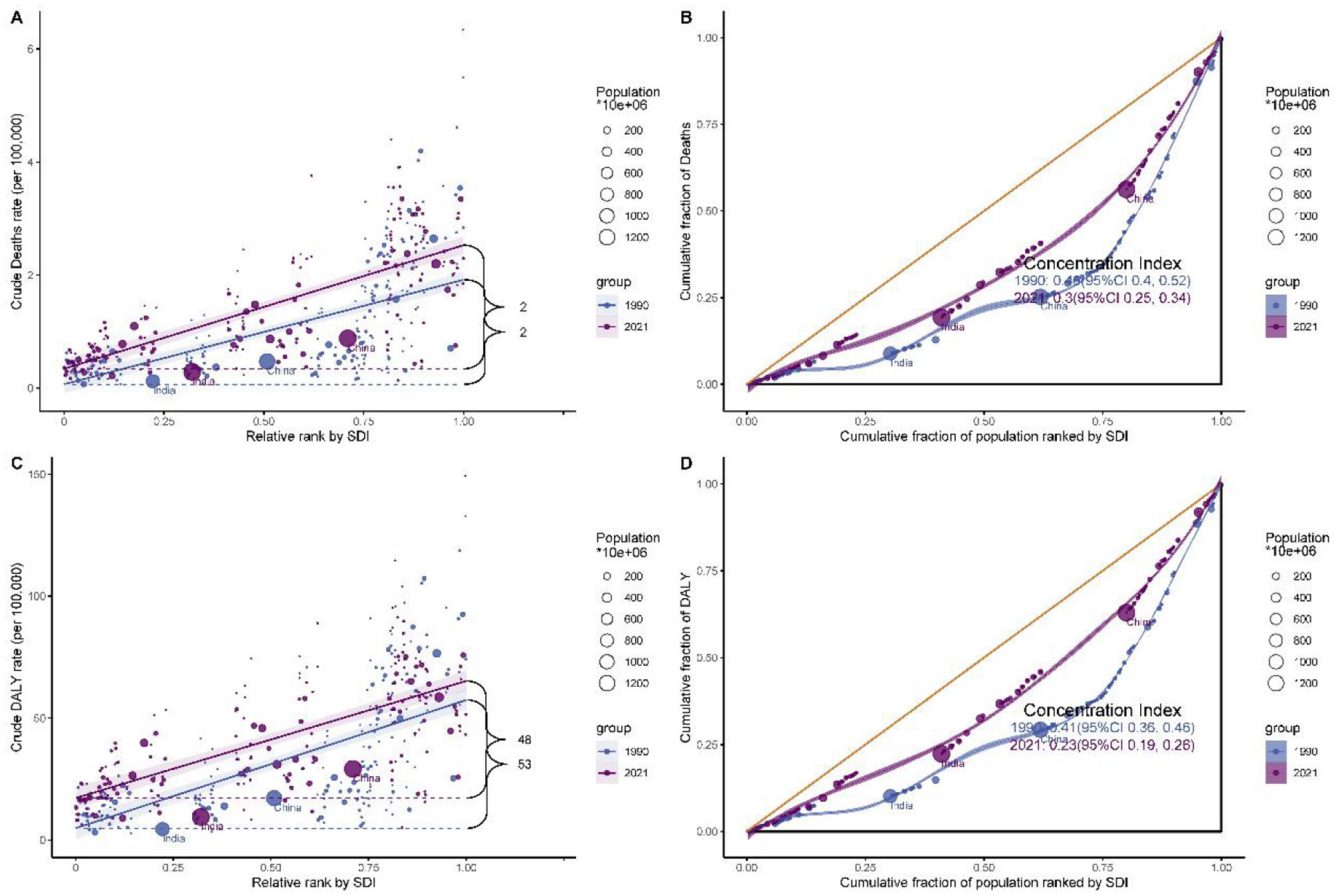
Health inequality analysis of breast cancer burden attributable to high red meat consumption. **(A)** Slope index of inequality for mortality. **(B)** Concentration Index for mortality. **(C)** Slope index of inequality for DALYs. **(D)** Concentration Index for DALYs.

Analysis of health inequalities in breast cancer burden from 1990 to 2021 revealed significant changes in global disease distribution (Figure 5 and Supplementary Table S12). The Slope Index (SI) and Concentration Index (CI) reflected absolute and relative inequalities, respectively. For mortality, the SI increased from 1.849 (95% CI: 1.539–2.159) in 1990 to 2.187 (95% CI: 1.907–2.467) in 2021, indicating a rise in absolute inequality. However, the CI declined from 0.461 (95% CI: 0.398–0.524) in 1990 to 0.297 (95% CI: 0.254–0.341) in 2021, suggesting a reduction in relative inequality. For DALYs, the SI peaked in 2003 at 58.595 (95% CI: 51.657–65.533) before gradually decreasing to 47.989 (95% CI: 40.747–55.232) in 2021. Similarly, the CI showed a consistent decline, from 0.412 (95% CI: 0.36–0.465) in 1990 to 0.229 (95% CI: 0.194–0.264) in 2021. These trends indicate that while absolute disparities in breast cancer burden increased during some periods, relative inequalities are progressively narrowing.

### Comparative assessment of breast cancer burden attributable to high red meat consumption across socio-demographic development levels

**Figure 6:**
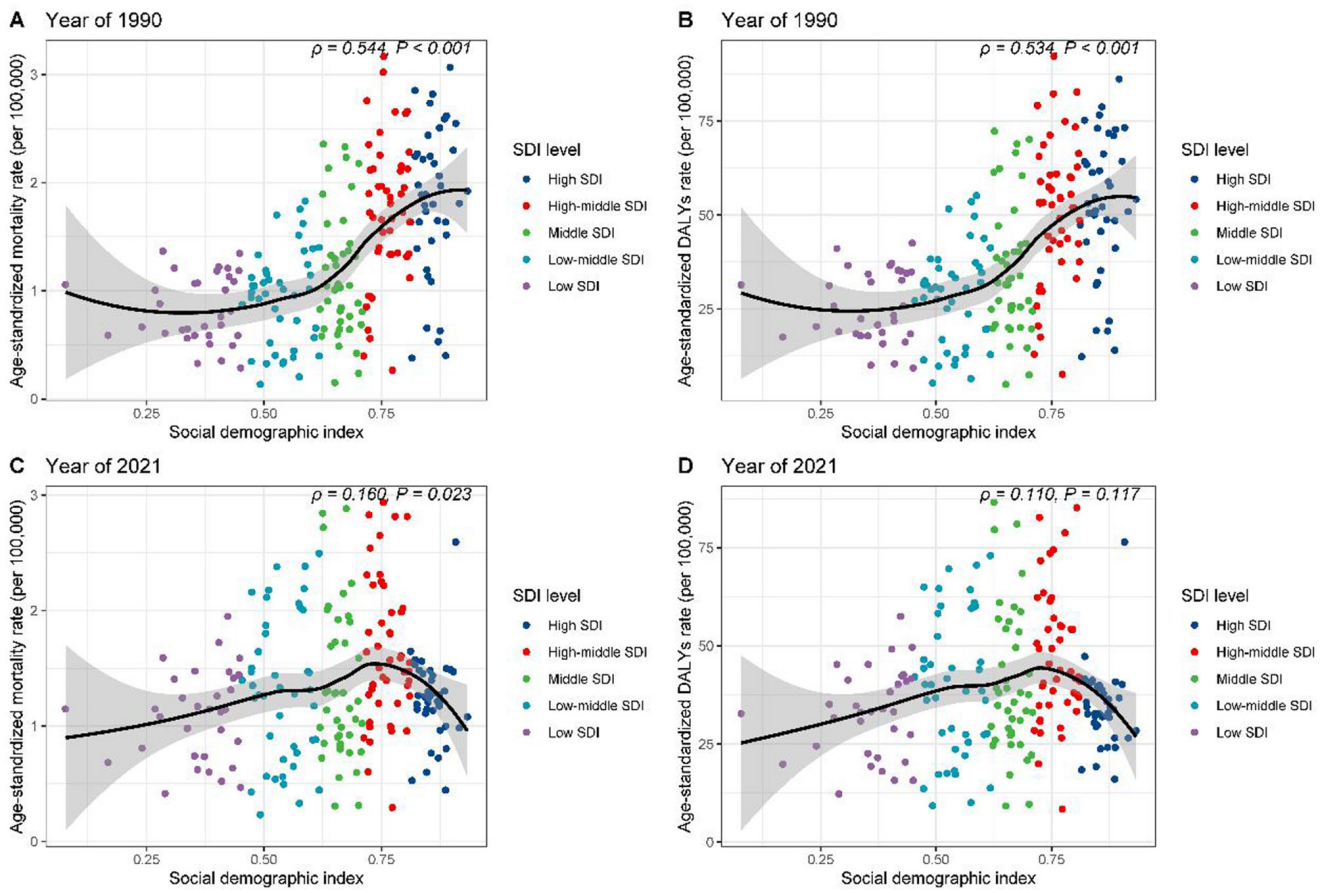
Correlation between SDI and age-standardized rates of breast cancer burden attributable to high red meat consumption across SDI quintiles. **(A)** SDI vs. age-standardized mortality rates, 1990. **(B)** SDI vs. age-standardized DALY rates, 1990. **(C)** SDI vs. age-standardized mortality rates, 2021. **(D)** SDI vs. age-standardized DALY rates, 2021.

The relationship between high red meat consumption and breast cancer burden among individuals aged 25 and above was explored in the context of socio-demographic development. In 1990, disease burden across five SDI regions showed an initial plateau followed by an upward trend, with ASMR (Figure 6A, *R* = 0.544, *p* < 0.001) and age-standardized DALY rates (Figure 6B, *R* = 0.534, *p* < 0.001) moderately positively correlated with SDI. However, in 2021, a different pattern emerged. The burden increased with rising SDI, peaking at an SDI of ~0.75, and then declined, with the correlation weakening (Figure 6C, *R* = 0.160, *p* = 0.023; Figure 6D, *R* = 0.110, *p* = 0.117).

Further regional analysis of 21 GBD regions confirmed these findings (Supplementary Figures S2A, B). High-income regions, such as North America and Southern Latin America, exhibited burdens significantly exceeding expectations based on SDI. At the national level, in 1990, countries such as Saint Kitts and Nevis and Palau had disease burdens markedly higher than expected (Supplementary Figures S3A, B). By 2021, Fiji and Palau stood out in terms of mortality rates (Supplementary Figure S3C), while Nauru and American Samoa showed elevated DALY rates (Supplementary Figure S3D). These results reveal the complex and dynamic relationship between high red meat consumption, socio-economic development, and breast cancer burden.

### Frontier analysis of breast cancer mortality and DALYs attributable to high red meat consumption: performance assessment across 204 countries

A frontier analysis of breast cancer mortality and disability-adjusted life years (DALYs) in females aged 25 and above across 204 countries and regions revealed significant global disparities in disease burden, closely associated with socio-economic development levels (Supplementary Figure S4 and Supplementary Tables S13, S14). In terms of mortality (Supplementary Table S13), nations such as Palau, Nauru, American Samoa, the Cook Islands, and the Bahamas exhibited actual mortality rates that far exceeded the levels predicted by their Socio-Demographic Index (SDI), with the largest deviation reaching 2.801. Similarly, for DALYs (Supplementary Table S14), Nauru, the Bahamas, Fiji, the Cook Islands, and Tonga showed the largest deviations from the frontier DALY values, with differences reaching as high as 81.473.

In contrast, countries such as Bangladesh, Oman, the Maldives, Sri Lanka, and India demonstrated relatively better performance in both mortality and DALY metrics. The actual values in these countries were close to or aligned with their expected SDI-based frontier values, indicating effective control of breast cancer disease burden. Notably, China ranked 23rd in both mortality and DALY deviations. The difference between actual and frontier values for mortality was 0.468 (Supplementary Table S13), while for DALYs, it was 14.808 (Supplementary Table S14). These findings suggest that China's breast cancer burden remains higher than expected based on its SDI, highlighting significant room for improvement in disease control and prevention.

This analysis underscores the global inequities in breast cancer burden, with certain high-risk regions far exceeding their expected burden relative to socio-economic development, while other nations have achieved relatively better outcomes through effective control measures.

### Machine learning models for predicting global breast cancer burden attributable to high red meat intake, 2022–2050

To enhance the accuracy and robustness of global breast cancer burden projections attributable to high red meat intake from 2022 to 2050, we utilized eight distinct machine learning algorithms: Prophet, ARIMA, TBATS, ElasticNet, ETS, VAR, Holt-Winters, and Theta (Supplementary Table S15). This comprehensive modeling approach aimed to capture diverse patterns and trends in the dataset, ensuring that projections account for both the total burden (number of deaths and DALYs) and age-standardized rates (ASRs). Our results revealed that different models performed optimally depending on the gender and measure (deaths or DALYs) being predicted, with detailed performance metrics comparison provided in Supplementary Table S16.

For male breast cancer attributable to high red meat intake, the ARIMA model consistently outperformed other methods. It exhibited the highest accuracy for predicting both the total number of deaths (MSE: 17.35, MAPE: 0.25%, *R*^2^: 0.998) and DALYs (MSE: 8,501.30, MAPE: 0.19%, *R*^2^: 0.999), as well as age-standardized death rates (MSE: 1.07 × 10^−8^, MAPE: 0.21%, *R*^2^: 0.97) and age-standardized DALY rates (MSE: 5.78 × 10^−6^, MAPE: 0.18%, *R*^2^: 0.98). By contrast, for female breast cancer attributable to high red meat intake, the Prophet model demonstrated superior performance in predicting the total number of deaths (MSE: 21,935.21, MAPE: 0.16%, *R*^2^: 0.999) and DALYs (MSE: 16,297,399.72, MAPE: 0.15%, *R*^2^: 0.999), as well as age-standardized death rates (MSE: 2.73 × 10^−5^, MAPE: 0.25%, *R*^2^: 0.92) and age-standardized DALY rates (MSE: 0.018, MAPE: 0.21%, *R*^2^: 0.84).

**Figure 7:**
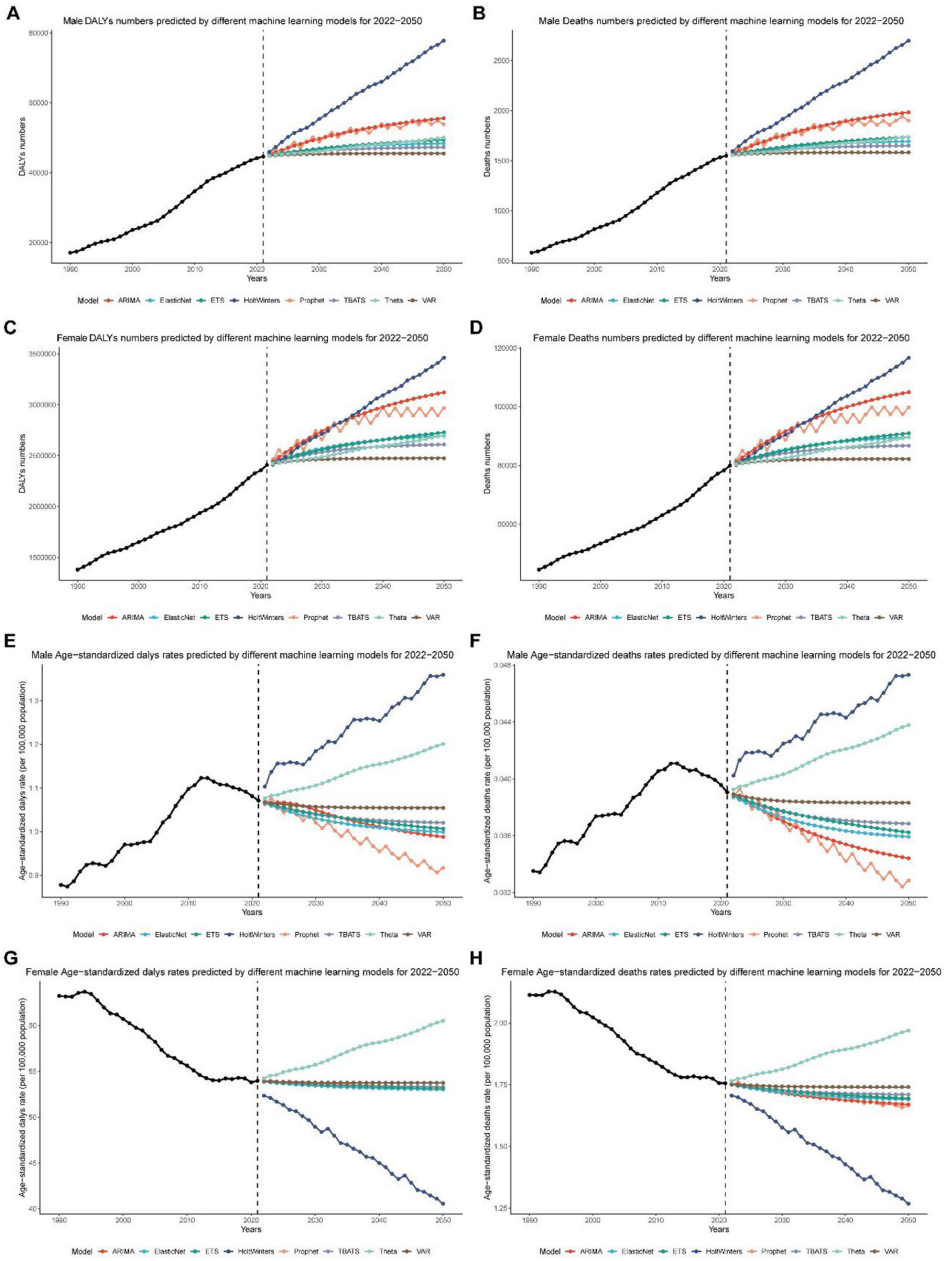
Global breast cancer burden projections (2022–2050) using machine learning models. **(A)** Projected global disability-adjusted life years (DALYs) for male breast cancer from 2022 to 2050, based on the ARIMA model. **(B)** Projected global deaths for male breast cancer from 2022 to 2050, based on the ARIMA model. **(C)** Projected global disability-adjusted life years (DALYs) for female breast cancer from 2022 to 2050, based on the Prophet model. **(D)** Projected global deaths for female breast cancer from 2022 to 2050, based on the Prophet model. **(E)** Projected global age-standardized disability-adjusted life year (DALY) rates for male breast cancer from 2022 to 2050, based on the ARIMA model. **(F)** Projected global age-standardized death rates for male breast cancer from 2022 to 2050, based on the ARIMA model. **(G)** Projected global age-standardized disability-adjusted life year (DALY) rates for female breast cancer from 2022 to 2050, based on the Prophet model. **(H)** Projected global age-standardized death rates for female breast cancer from 2022 to 2050, based on the Prophet model.

Based on these results, ARIMA was selected for all male projections and Prophet for all female projections. By 2050, the ARIMA model forecasts that global male breast cancer deaths attributable to high red meat intake will rise to 1,982 cases, DALYs to 55,585, the age-standardized death rate (ASR) to 0.0344 deaths per 100,000, and the DALY ASR to 0.9876 per 100,000 (Figures 7A, B, E, F and Tables 1, 2). For females, the Prophet model predicts 99,749 deaths, 2,965,835 DALYs, an ASR of 1.6655 deaths per 100,000, and a DALY ASR of 53.1882 per 100,000 by 2050 (Figures 7C, D, G, H and Tables 1, 2).

**Table 1:** Projected trends in absolute numbers of DALYs and deaths from breast cancer attributable to high red meat intake for males and females from 2022 to 2050 using eight machine learning algorithms.

**Sex**	**location**	**year**	**Deaths**								**DALYs**							
			**Number of cases**								**Number of cases**							
			**Prophet**	**ARIMA**	**TBATS**	**ElasticNet**	**ETS**	**VAR**	**HoltWinters**	**Theta**	**Prophet**	**ARIMA**	**TBATS**	**ElasticNet**	**ETS**	**VAR**	**HoltWinters**	**Theta**
Male	Global	2022	1,583	1,572	1,559	1,561	1,563	1,555	1,596	1,557	45,475	45,192	44,894	44,948	44,986	44,787	45,965	44,850
Male	Global	2023	1,637	1,598	1,569	1,573	1,575	1,560	1,644	1,566	46,848	45,865	45,137	45,245	45,286	44,919	47,344	45,100
Male	Global	2024	1,584	1,623	1,577	1,583	1,586	1,564	1,693	1,567	45,495	46,505	45,357	45,519	45,563	45,027	48,764	45,135
Male	Global	2025	1,655	1,647	1,585	1,593	1,596	1,568	1,740	1,574	47,291	47,114	45,555	45,772	45,821	45,115	50,227	45,350
Male	Global	2026	1,707	1,670	1,591	1,602	1,605	1,571	1,782	1,580	48,607	47,693	45,735	46,006	46,061	45,187	51,418	45,511
Male	Global	2027	1,655	1,692	1,598	1,610	1,614	1,573	1,808	1,585	47,309	48,243	45,898	46,222	46,286	45,247	52,153	45,649
Male	Global	2028	1,714	1,713	1,603	1,617	1,622	1,575	1,834	1,589	48,870	48,767	46,045	46,421	46,497	45,295	52,857	45,762
Male	Global	2029	1,764	1,732	1,608	1624	1,630	1,576	1,874	1,594	50,134	49,266	46,178	46,605	46,697	45,335	54,010	45,911
Male	Global	2030	1,715	1,751	1,613	1,631	1,637	1,577	1,918	1,600	48,886	49,740	46,298	46,774	46,887	45,367	55,354	46,079
Male	Global	2031	1,763	1,769	1,617	1,637	1,644	1,578	1,954	1,607	50,206	50,191	46,407	46,931	47,067	45,394	56,456	46,282
Male	Global	2032	1,811	1,786	1,621	1,642	1,651	1,579	2,001	1,615	51,422	50,620	46,506	47,075	47,238	45,416	57,840	46,506
Male	Global	2033	1,764	1,802	1,624	1,647	1,657	1,580	2,031	1,623	50,221	51,028	46,595	47,209	47,402	45,433	58,697	46,763
Male	Global	2034	1,811	1,817	1,627	1,652	1,663	1,580	2,074	1,632	51,408	51,416	46,676	47,332	47,559	45,448	59,942	47,006
Male	Global	2035	1,857	1,832	1,630	1,656	1,669	1,581	2,121	1,640	52,579	51,785	46,749	47,446	47,709	45,460	61,295	47,240
Male	Global	2036	1,811	1,846	1,632	1,660	1,675	1,581	2,166	1,648	51,421	52,136	46,815	47,551	47,853	45,470	62,598	47,465
Male	Global	2037	1,857	1,859	1,635	1,664	1,680	1,582	2,198	1,656	52,566	52,470	46,875	47,648	47,991	45,478	63,428	47,693
Male	Global	2038	1,812	1,872	1,637	1,667	1,685	1,582	2,240	1,662	51,434	52,788	46,929	47,737	48,125	45,484	64,586	47,866
Male	Global	2039	1,856	1,883	1,639	1,671	1,690	1,582	2,266	1,667	52,741	53,090	46,978	47,820	48,253	45,490	65,320	48,007
Male	Global	2040	1,900	1,895	1,640	1,674	1,695	1,582	2,291	1,671	53,848	53,378	47,022	47,896	48,377	45,494	66,025	48,123
Male	Global	2041	1,857	1,906	1,642	1,676	1,700	1,582	2,332	1,675	52,753	53,651	47,063	47,966	48,497	45,498	67,177	48,259
Male	Global	2042	1,899	1,916	1,643	1,679	1,704	1,582	2,376	1,681	53,836	53,912	47,099	48,031	48,613	45,501	68,521	48,417
Male	Global	2043	1,857	1,926	1,644	1,681	1,709	1,583	2,412	1,687	52,764	54,159	47,132	48,091	48,726	45,503	69,624	48,601
Male	Global	2044	1,899	1,935	1,646	1,683	1,713	1,583	2,459	1,694	53,826	54,395	47,161	48,147	48,835	45,505	71,008	48,781
Male	Global	2045	1,857	1,944	1,647	1,685	1,717	1,583	2,488	1,699	54,877	54,619	47,188	48,198	48,941	45,507	71,864	48,950
Male	Global	2046	1,899	1,952	1,648	1,687	1,721	1,583	2,532	1,706	53,836	54,832	47,213	48,245	49,044	45,508	73,110	49,148
Male	Global	2047	1,858	1,960	1,648	1,688	1,725	1,583	2,579	1,714	54,867	55,034	47,235	48,288	49,144	45,509	74,462	49,373
Male	Global	2048	1,899	1,968	1,649	1,690	1,729	1,583	2,624	1,723	53,845	55,227	47,254	48,329	49,241	45,510	75,765	49,618
Male	Global	2049	1,939	1,975	1,650	1,691	1,732	1,583	2,656	1,730	54,858	55,410	47,273	48,366	49,336	45,511	76,595	49,824
Male	Global	2050	1,899	1,982	1,650	1,693	1,736	1,583	2,698	1,736	53,854	55,585	47,289	48,400	49,428	45,511	77,753	50,002
Female	Global	2022	81,894	81,248	80,642	80,779	80,782	80,368	80,163	80,358	2,462,336	2,443,921	2,427,430	2,431,497	2,431,053	2,419,295	2,410,493	2,419,160
Female	Global	2023	85,028	82,784	81,262	81,538	81469	80,705	81,687	80,806	2,551,728	2,487,713	2,445,832	2,454,026	2,451,020	2,429,285	2,453,386	2,432,659
Female	Global	2024	81,939	84,244	81,823	82,239	82,104	80,980	82,981	80,881	2,463,617	2,529,368	2,462,483	2,474,823	2,469,451	2,437,465	2,489,576	2,434,983
Female	Global	2025	85,407	85,634	82,330	82,885	82,693	81,206	84,343	81,269	2,561,137	2,568,992	2,477,550	2,494,021	2,486,565	2,444,162	2,528,896	2,446,674
Female	Global	2026	88,411	86,955	82,790	83,482	83,243	81,390	86,024	81,558	2,646,797	2,606,683	2,491,182	2,511,742	2,502,539	2,449,645	2,578,224	2,455,361
Female	Global	2027	85,449	88,212	83,205	84,033	83,759	81,542	87,378	81,801	2,562,308	2,642,537	2,503,518	2,528,102	2,517,514	2,454,134	2,619,954	2,462,661
Female	Global	2028	88,730	89,408	83,581	84,542	84,244	81,665	88,374	81,998	2,658,096	2,676,641	2,514,679	2,543,203	2,531,609	2,457,809	2,651,174	2,468,555
Female	Global	2029	91,614	90,546	83,921	85,012	84,703	81,767	89,505	82,258	2,740,346	2,709,083	2,524,779	2,557,144	2,544,920	2,460,818	2,685,221	2,476,375
Female	Global	2030	88,768	91,628	84,229	85,445	85,137	81,850	90,457	82,555	2,659,166	2,739,942	2,533,917	2,570,012	2,557,531	2,463,282	2,711,417	2,485,290
Female	Global	2031	91,578	92,657	84,508	85,845	85,549	81,918	91,610	82,917	2,739,307	2,769,296	2,542,186	2,581,891	2,569,511	2,465,299	2,742,781	2,496,157
Female	Global	2032	94,353	93,636	84,760	86,215	85,942	81,973	93,548	83,319	2,818,440	2,797,218	2,549,667	2,592,857	2,580,921	2,466,951	2,797,447	2,508,256
Female	Global	2033	91,612	94,567	84,988	86,556	86,317	82,019	94,502	83,780	2,740,285	2,823,779	2,556,437	2,602,980	2,591,812	2,468,303	2,825,270	2,522,174
Female	Global	2034	94,319	95,453	85,194	86,870	86,676	82,056	95,502	84,220	2,817,491	2,849,045	2,562,563	2,612,325	2,602,229	2,469,410	2,851,839	2,535,436
Female	Global	2035	91,645	96,296	85,381	87,161	87,020	82,087	97,025	84,644	2,893,776	2,873,078	2,568,105	2,620,951	2,612,213	2,470,316	2,894,732	2,548,225
Female	Global	2036	94,709	97,097	85,550	87,429	87,350	82,112	98,320	85,050	2,818,385	2,895,939	2,573,121	2,628,914	2,621,797	2,471,058	2,930,922	2,560,465
Female	Global	2037	97,322	97,860	85,703	87,677	87,667	82,132	99,681	85,458	2,892,908	2,917,685	2,577,658	2,636,265	2,631,012	2,471,666	2,970,242	2,572,733
Female	Global	2038	94,738	98,585	85,841	87,905	87,973	82,149	101,363	85,766	2,819,227	2,938,370	2,581,765	2,643,050	2,639,887	2,472,163	3,019,570	2,581,992
Female	Global	2039	97,293	99,275	85,966	88,116	88,267	82,163	102,717	86,013	2,892,091	2,958,047	2,585,480	2,649,314	2,648,444	2,472,570	3,061,299	2,589,407
Female	Global	2040	94,766	99,931	86,079	88,311	88,552	82,174	103,713	86,216	2,964,162	2,976,764	2,588,842	2,655,096	2,656,706	2,472,904	3,092,520	2,595,494
Female	Global	2041	97,266	100,556	86,182	88,491	88,827	82,183	104,844	86,455	2,892,860	2,994,568	2,591,884	2,660,434	2,664,693	2,473,177	3,126,567	2,602,641
Female	Global	2042	94,792	101,150	86,275	88,657	89,093	82,191	105,796	86,733	2,963,416	3,011,504	2,594,636	2,665,361	2,672,422	2,473,400	3,152,762	2,610,990
Female	Global	2043	97,391	101,714	86,358	88,810	89,351	82,197	106,949	87,062	2,893,585	3,027,614	2,597,126	2,669,910	2,679,910	2,473,583	3,184,126	2,620,914
Female	Global	2044	99,814	102,252	86,434	88,952	89,601	82,202	108,887	87,388	2,962,712	3,042,938	2,599,380	2,674,108	2,687,170	2,473,733	3,238,793	2,630,742
Female	Global	2045	97,415	102,763	86,503	89,082	89,843	82,206	109,841	87,696	2,894,268	3,057,515	2,601,419	2,677,984	2,694,218	2,473,856	3,266,616	2,640,030
Female	Global	2046	99,791	103,249	86,565	89,203	90,079	82,209	110,841	88,058	2,962,050	3,071,381	2,603,264	2,681,562	2,701,063	2,473,956	3,293,185	2,650,972
Female	Global	2047	97,437	103,712	86,621	89,314	90,308	82,212	112,364	88,468	2,894,910	3,084,570	2,604,933	2,684,865	2,707,719	2,474,038	3,336,077	2,663,365
Female	Global	2048	99,769	104,151	86,672	89,417	90,531	82,214	113,659	88,913	2,961,426	3,097,117	2,606,444	2,687,914	2,714,195	2,474,106	3,372,268	2,676,786
Female	Global	2049	97,458	104,570	86,718	89,511	90,748	82,216	115,020	89,285	2,895,516	3,109,051	2,607,811	2,690,729	2,720,500	2,474,161	3,411,587	2,687,991
Female	Global	2050	99,749	104,968	86,760	89,599	90,960	82,218	116,702	89,603	2,965,835	3,120,404	2,609,047	2,693,327	2,726,644	2,474,206	3,460,916	2,697,565

**Table 2:** Projected trends in age-standardized rates of DALYs and deaths from breast cancer attributable to high red meat intake for males and females from 2022 to 2050 using eight machine learning algorithms.

**Sex**	**location**	**year**	**Deaths**								**DALYs**							
			**Number of ASR**								**Number of ASR**							
			**Prophet**	**ARIMA**	**TBATS**	**ElasticNet**	**ETS**	**VAR**	**HoltWinters**	**Theta**	**Prophet**	**ARIMA**	**TBATS**	**ElasticNet**	**ETS**	**VAR**	**HoltWinters**	**Theta**
Male	Global	2022	0.0387	0.0388	0.0388	0.0388	0.0388	0.0389	0.0402	0.0392	1.0620	1.0658	1.0658	1.0648	1.0661	1.0679	1.1031	1.0763
Male	Global	2023	0.0393	0.0385	0.0386	0.0385	0.0387	0.0388	0.0413	0.0395	1.0765	1.0661	1.0612	1.0592	1.0621	1.0654	1.1362	1.0823
Male	Global	2024	0.0385	0.0384	0.0384	0.0383	0.0385	0.0387	0.0418	0.0395	1.0576	1.0669	1.0570	1.0539	1.0583	1.0633	1.1559	1.0838
Male	Global	2025	0.0379	0.0382	0.0383	0.0381	0.0384	0.0386	0.0418	0.0397	1.0436	1.0663	1.0533	1.0491	1.0549	1.0616	1.1552	1.0891
Male	Global	2026	0.0385	0.0379	0.0381	0.0379	0.0382	0.0386	0.0419	0.0399	1.0574	1.0646	1.0498	1.0447	1.0517	1.0603	1.1588	1.0929
Male	Global	2027	0.0377	0.0377	0.0380	0.0377	0.0381	0.0385	0.0418	0.0400	1.0390	1.0619	1.0467	1.0406	1.0486	1.0591	1.1571	1.0960
Male	Global	2028	0.0371	0.0374	0.0379	0.0376	0.0380	0.0385	0.0416	0.0401	1.0256	1.0583	1.0439	1.0368	1.0458	1.0582	1.1534	1.0985
Male	Global	2029	0.0377	0.0372	0.0378	0.0374	0.0378	0.0385	0.0420	0.0402	1.0388	1.0540	1.0414	1.0333	1.0431	1.0575	1.1667	1.1018
Male	Global	2030	0.0369	0.0370	0.0377	0.0373	0.0377	0.0384	0.0425	0.0403	1.0205	1.0490	1.0391	1.0300	1.0405	1.0568	1.1845	1.1056
Male	Global	2031	0.0364	0.0368	0.0376	0.0371	0.0376	0.0384	0.0426	0.0405	1.0075	1.0443	1.0370	1.0271	1.0381	1.0563	1.1930	1.1103
Male	Global	2032	0.0369	0.0366	0.0375	0.0370	0.0375	0.0384	0.0430	0.0407	1.0203	1.0398	1.0351	1.0243	1.0358	1.0559	1.2064	1.1157
Male	Global	2033	0.0361	0.0364	0.0374	0.0369	0.0374	0.0384	0.0428	0.0409	1.0021	1.0355	1.0334	1.0218	1.0336	1.0556	1.2046	1.1220
Male	Global	2034	0.0356	0.0363	0.0374	0.0368	0.0373	0.0384	0.0433	0.0411	0.9896	1.0314	1.0319	1.0194	1.0315	1.0553	1.2196	1.1281
Male	Global	2035	0.0361	0.0361	0.0373	0.0367	0.0372	0.0384	0.0440	0.0413	1.0019	1.0276	1.0305	1.0172	1.0295	1.0551	1.2390	1.1339
Male	Global	2036	0.0354	0.0359	0.0372	0.0366	0.0372	0.0384	0.0445	0.0416	0.9840	1.0239	1.0293	1.0152	1.0276	1.0549	1.2561	1.1394
Male	Global	2037	0.0349	0.0358	0.0372	0.0366	0.0371	0.0383	0.0445	0.0417	0.9720	1.0204	1.0281	1.0134	1.0257	1.0547	1.2554	1.1449
Male	Global	2038	0.0354	0.0357	0.0371	0.0365	0.0370	0.0383	0.0446	0.0419	0.9839	1.0170	1.0271	1.0117	1.0239	1.0546	1.2590	1.1489
Male	Global	2039	0.0347	0.0355	0.0371	0.0364	0.0369	0.0383	0.0445	0.0420	0.9663	1.0138	1.0262	1.0101	1.0222	1.0545	1.2573	1.1521
Male	Global	2040	0.0342	0.0354	0.0371	0.0363	0.0369	0.0383	0.0443	0.0421	0.9547	1.0108	1.0253	1.0087	1.0205	1.0544	1.2536	1.1546
Male	Global	2041	0.0347	0.0353	0.0370	0.0363	0.0368	0.0383	0.0447	0.0422	0.9662	1.0079	1.0245	1.0073	1.0189	1.0544	1.2669	1.1576
Male	Global	2042	0.0341	0.0352	0.0370	0.0362	0.0367	0.0383	0.0452	0.0423	0.9492	1.0052	1.0239	1.0061	1.0173	1.0543	1.2847	1.1612
Male	Global	2043	0.0336	0.0350	0.0370	0.0362	0.0367	0.0383	0.0453	0.0425	0.9379	1.0026	1.0232	1.0050	1.0158	1.0543	1.2932	1.1656
Male	Global	2044	0.0340	0.0349	0.0370	0.0361	0.0366	0.0383	0.0457	0.0427	0.9491	1.0001	1.0227	1.0039	1.0143	1.0542	1.3066	1.1700
Male	Global	2045	0.0334	0.0348	0.0369	0.0361	0.0365	0.0383	0.0455	0.0428	0.9327	0.9978	1.0222	1.0029	1.0129	1.0542	1.3048	1.1744
Male	Global	2046	0.0330	0.0347	0.0369	0.0361	0.0365	0.0383	0.0460	0.0430	0.9218	0.9956	1.0217	1.0020	1.0115	1.0542	1.3198	1.1795
Male	Global	2047	0.0334	0.0347	0.0369	0.0360	0.0364	0.0383	0.0467	0.0432	0.9326	0.9934	1.0213	1.0012	1.0102	1.0541	1.3392	1.1852
Male	Global	2048	0.0329	0.0346	0.0369	0.0360	0.0364	0.0383	0.0472	0.0434	0.9170	0.9914	1.0209	1.0004	1.0089	1.0541	1.3563	1.1913
Male	Global	2049	0.0324	0.0345	0.0369	0.0360	0.0363	0.0383	0.0472	0.0436	0.9063	0.9895	1.0205	0.9997	1.0076	1.0541	1.3556	1.1964
Male	Global	2050	0.0329	0.0344	0.0368	0.0359	0.0362	0.0383	0.0473	0.0438	0.9169	0.9876	1.0202	0.9991	1.0064	1.0541	1.3592	1.2007
Female	Global	2022	1.7491	1.7513	1.7511	1.7502	1.7511	1.7530	1.7059	1.7646	53.8968	53.9154	53.8860	53.8727	53.8907	53.9126	52.3340	54.2230
Female	Global	2023	1.7599	1.7461	1.7470	1.7451	1.7473	1.7507	1.6993	1.7744	53.9870	53.8712	53.8258	53.7990	53.8392	53.8799	52.0905	54.5256
Female	Global	2024	1.7483	1.7411	1.7432	1.7404	1.7437	1.7489	1.6845	1.7773	53.8872	53.8292	53.7713	53.7309	53.7916	53.8532	51.6698	54.6133
Female	Global	2025	1.7378	1.7363	1.7398	1.7360	1.7404	1.7473	1.6714	1.7861	53.7995	53.7892	53.7220	53.6681	53.7474	53.8313	51.3264	54.8830
Female	Global	2026	1.7481	1.7318	1.7367	1.7320	1.7373	1.7461	1.6514	1.7924	53.8860	53.7511	53.6774	53.6101	53.7062	53.8133	50.7887	55.0762
Female	Global	2027	1.7370	1.7275	1.7339	1.7283	1.7344	1.7451	1.6427	1.7974	53.7984	53.7150	53.6370	53.5566	53.6675	53.7986	50.6501	55.2300
Female	Global	2028	1.7270	1.7234	1.7313	1.7248	1.7316	1.7442	1.6194	1.8012	53.7143	53.6805	53.6005	53.5072	53.6312	53.7866	50.1087	55.3469
Female	Global	2029	1.7368	1.7195	1.7290	1.7217	1.7291	1.7436	1.6011	1.8063	53.7973	53.6478	53.5675	53.4616	53.5968	53.7768	49.6601	55.5059
Female	Global	2030	1.7259	1.7158	1.7270	1.7188	1.7266	1.7430	1.5759	1.8124	53.7100	53.6167	53.5376	53.4194	53.5642	53.7687	48.9279	55.6926
Female	Global	2031	1.7163	1.7123	1.7251	1.7161	1.7243	1.7425	1.5572	1.8201	53.6291	53.5870	53.5105	53.3806	53.5333	53.7621	48.3676	55.9293
Female	Global	2032	1.7258	1.7089	1.7234	1.7136	1.7221	1.7422	1.5682	1.8289	53.7090	53.5589	53.4860	53.3447	53.5039	53.7567	48.7274	56.2012
Female	Global	2033	1.7151	1.7057	1.7218	1.7113	1.7200	1.7419	1.5396	1.8393	53.6220	53.5321	53.4639	53.3116	53.4758	53.7523	47.9576	56.5206
Female	Global	2034	1.7059	1.7027	1.7204	1.7091	1.7180	1.7416	1.5141	1.8494	53.5441	53.5066	53.4438	53.2810	53.4489	53.7486	47.1589	56.8293
Female	Global	2035	1.7150	1.6998	1.7192	1.7072	1.7160	1.7414	1.5076	1.8591	53.6211	53.4823	53.4257	53.2528	53.4231	53.7457	46.9724	57.1273
Female	Global	2036	1.7046	1.6971	1.7180	1.7054	1.7142	1.7412	1.4927	1.8683	53.5346	53.4592	53.4093	53.2267	53.3984	53.7433	46.5516	57.4091
Female	Global	2037	1.6957	1.6945	1.7170	1.7037	1.7124	1.7411	1.4796	1.8773	53.4593	53.4373	53.3944	53.2026	53.3746	53.7413	46.2083	57.6848
Female	Global	2038	1.7045	1.6920	1.7161	1.7022	1.7107	1.7410	1.4597	1.8838	53.5337	53.4164	53.3810	53.1804	53.3517	53.7396	45.6706	57.8855
Female	Global	2039	1.6944	1.6896	1.7152	1.7007	1.7090	1.7409	1.4509	1.8887	53.4474	53.3966	53.3688	53.1599	53.3296	53.7383	45.5320	58.0385
Female	Global	2040	1.6857	1.6874	1.7145	1.6994	1.7074	1.7408	1.4276	1.8926	53.3747	53.3777	53.3578	53.1410	53.3082	53.7372	44.9905	58.1587
Female	Global	2041	1.6943	1.6852	1.7138	1.6982	1.7059	1.7408	1.4094	1.8973	53.4466	53.3597	53.3479	53.1236	53.2876	53.7363	44.5420	58.3037
Female	Global	2042	1.6845	1.6832	1.7132	1.6971	1.7044	1.7407	1.3841	1.9030	53.3607	53.3426	53.3389	53.1074	53.2677	53.7356	43.8097	58.4810
Female	Global	2043	1.6761	1.6813	1.7126	1.6961	1.7029	1.7407	1.3655	1.9102	53.2902	53.3264	53.3307	53.0925	53.2483	53.7350	43.2494	58.7019
Female	Global	2044	1.6844	1.6794	1.7121	1.6951	1.7015	1.7406	1.3765	1.9177	53.3600	53.3109	53.3233	53.0788	53.2296	53.7345	43.6093	58.9299
Female	Global	2045	1.6748	1.6777	1.7116	1.6942	1.7002	1.7406	1.3479	1.9250	53.2745	53.2962	53.3167	53.0661	53.2114	53.7341	42.8395	59.1536
Female	Global	2046	1.6667	1.6760	1.7112	1.6934	1.6988	1.7406	1.3223	1.9337	53.2061	53.2822	53.3106	53.0544	53.1937	53.7338	42.0408	59.4189
Female	Global	2047	1.6748	1.6744	1.7108	1.6927	1.6975	1.7406	1.3158	1.9434	53.2738	53.2689	53.3052	53.0436	53.1766	53.7335	41.8542	59.7167
Female	Global	2048	1.6655	1.6729	1.7105	1.6920	1.6963	1.7405	1.3010	1.9537	53.1887	53.2562	53.3002	53.0336	53.1598	53.7333	41.4335	60.0331
Female	Global	2049	1.6576	1.6715	1.7102	1.6913	1.6951	1.7405	1.2878	1.9622	53.1222	53.2442	53.2958	53.0244	53.1436	53.7331	41.0902	60.2919
Female	Global	2050	1.6655	1.6701	1.7099	1.6907	1.6939	1.7405	1.2679	1.9691	53.1882	53.2327	53.2917	53.0159	53.1277	53.7330	40.5525	60.5050

These findings demonstrate the strong performance and reliability of models such as ARIMA and Prophet in forecasting both the total burden and age-standardized rates of global breast cancer attributable to high red meat intake. The projections generated by these models provide a robust evidence base for guiding future health policies, optimizing resource allocation, and developing targeted strategies to address the global breast cancer burden associated with dietary risk factors.

### Association between health workforce density and red meat-induced breast cancer burden: a global analysis

**Figure 8:**
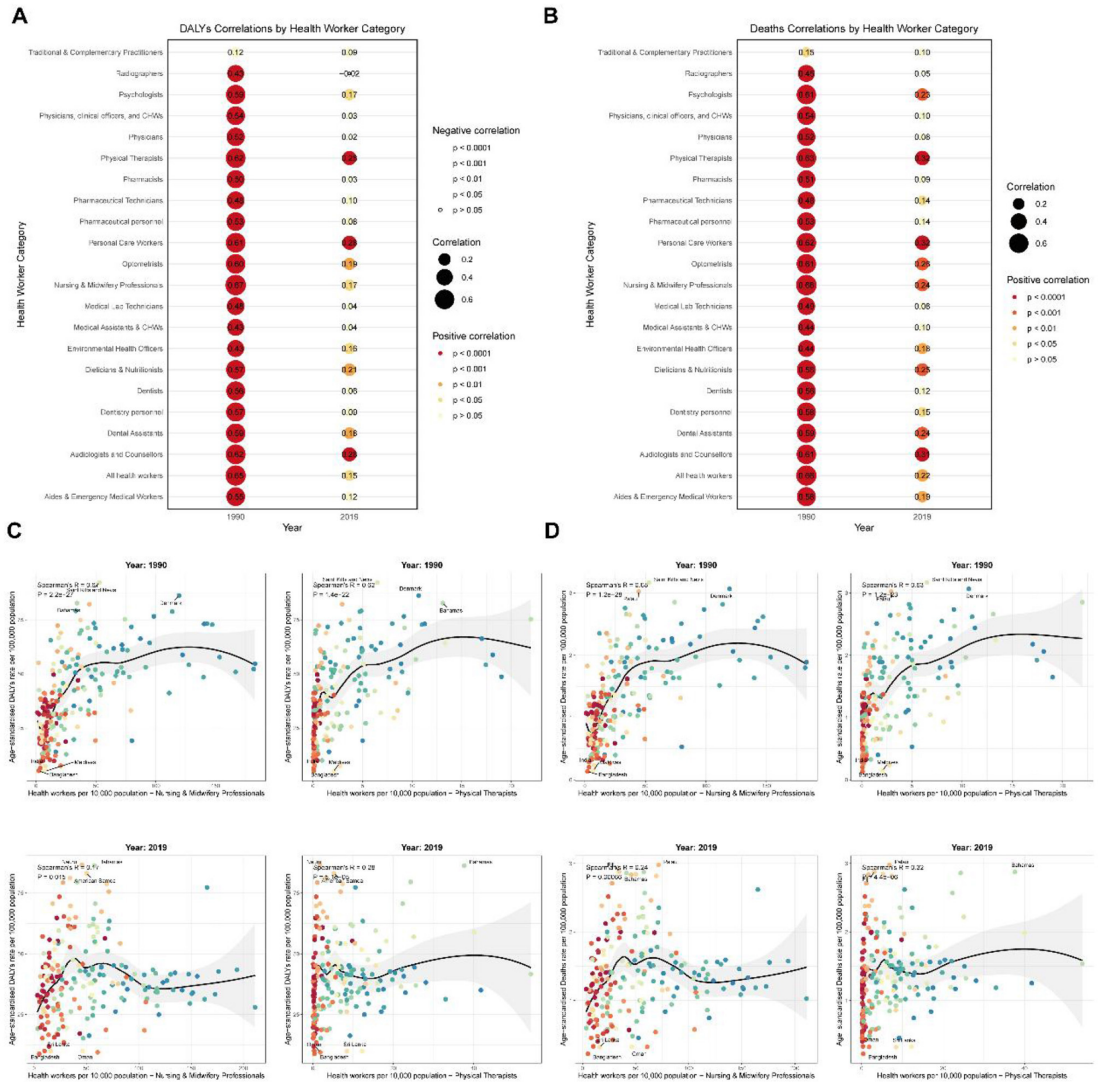
Global correlations between health workforce density and red meat-induced breast cancer burden (1990 and 2019). **(A)** Correlations between health workforce categories and disability-adjusted life years (DALYs) due to red meat-induced breast cancer in 1990 and 2019, with point size representing correlation strength and color indicating significance level. **(B)** Correlations between health workforce categories and deaths due to red meat-induced breast cancer in 1990 and 2019, with point size representing correlation strength and color indicating significance level. **(C)** Scatter plots showing the relationship between the density of Nursing and Midwifery Professionals and Physical Therapists (per 10,000 population) and DALYs due to red meat-induced breast cancer in 1990 and 2019, with labeled extreme values. **(D)** Scatter plots showing the relationship between the density of Nursing and Midwifery Professionals and Physical Therapists (per 10,000 population) and deaths due to red meat-induced breast cancer in 1990 and 2019, with labeled extreme values.

Our analysis revealed significant associations between health workforce density and the burden of red meat-induced breast cancer across 204 countries and territories. In 1990, strong positive correlations were observed between all health worker categories and both mortality and disability metrics, with particularly robust associations found for Nursing and Midwifery Professionals (Deaths: *r* = 0.68, *p* < 1.20E−28; DALYs: *r* = 0.67, *p* < 2.20E−27) and Physical Therapists (Deaths: *r* = 0.63, *p* < 1.20E−23; DALYs: *r* = 0.62, *p* < 1.40E−22) (Figures 8A, B). By 2019, the strength of these correlations had substantially attenuated, though significant positive associations persisted for select health workforce categories, notably Physical Therapists (Deaths: *r* = 0.32, *p* < 4.40E−06; DALYs: *r* = 0.28, *p* < 5.10E−05) and Nursing and Midwifery Professionals (Deaths: *r* = 0.24, *p* = 0.00066; DALYs: *r* = 0.17, *p* = 0.015).

Further examination of country-level data for these two critical workforce categories revealed pronounced disparities in breast cancer burden relative to healthcare workforce density (Figures 8C, D). In 1990, high-income nations such as Saint Kitts and Nevis, Denmark, and Palau exhibited both elevated healthcare workforce density and breast cancer mortality rates, with death rates exceeding 3.0 per 100,000 population, while countries with minimal healthcare infrastructure such as Bangladesh (Nursing density: 2.177 per 10,000; Deaths: 0.134 per 100,000) and Maldives (Nursing density: 7.112 per 10,000; Deaths: 0.149 per 100,000) reported substantially lower disease burden. By 2019, while the correlation pattern persisted, several notable shifts emerged: Palau, Fiji, and the Bahamas exhibited the highest mortality rates (>2.8 per 100,000) despite varying healthcare workforce densities, while countries such as Oman demonstrated relatively robust healthcare systems (Nursing density: 60.859 per 10,000) yet maintained comparatively low mortality rates (0.323 per 100,000).

This temporal evolution in associations between health workforce density and breast cancer burden suggests complex interactions between healthcare system development, disease detection capabilities, and population risk factors. The weakening correlation from 1990 to 2019 indicates that while healthcare workforce expansion may initially coincide with increased documented disease burden through improved detection, long-term investments in healthcare systems and preventive strategies can ultimately contribute to disease mitigation, particularly in regions that have implemented targeted public health interventions addressing modifiable risk factors such as red meat consumption.

## Discussion

Our analysis reveals a significant increase in the global burden of breast cancer from 1990 to 2021, with notable gender disparities. While overall age-standardized mortality rates decreased, the absolute number of deaths and DALYs increased substantially, primarily driven by population growth and aging. This trend aligns with previous studies highlighting the growing global cancer burden (7, 27). Interestingly, our findings show a slight increase in male breast cancer mortality rates, contrasting with the decreasing trend in females. This gender disparity may be attributed to differences in awareness, screening practices, and biological factors (28). The increasing male breast cancer burden, particularly in certain age groups, underscores the need for targeted interventions and increased awareness of this rare but significant condition (29).

The analysis across SDI quintiles reveals a complex relationship between socio-economic development and breast cancer burden. High-SDI regions demonstrated significant decreases in mortality and DALY rates, while low-middle SDI regions experienced increases. This pattern reflects the “cancer transition” phenomenon, where cancer burden shifts from infection-related cancers to those associated with westernized lifestyles as countries develop (30). The regional disparities observed, with reductions in high-income regions and increases in regions like North Africa, Middle East, and South Asia, further support this concept. These findings highlight the need for tailored prevention and control strategies that consider a region's stage of epidemiological transition (31).

The age-specific analysis reveals concerning trends, particularly the increase in mortality and DALYs for younger age groups (25–34 years). This aligns with recent studies reporting a rising incidence of early-onset breast cancer in many countries (32). The age-period-cohort analysis further elucidates these trends, showing a downward cohort effect for younger birth cohorts. This suggests that while overall risk is decreasing for more recent generations, other factors may be contributing to the increased burden in young adults. These findings emphasize the importance of understanding risk factors specific to younger populations and potentially reconsidering screening guidelines for high-risk groups (33, 34).

While our study focused on the potential impact of high red meat consumption on breast cancer burden, the relationship appears complex and multifaceted. Recent comprehensive meta-analyses have provided robust evidence supporting this relationship, with studies demonstrating that high red meat intake is associated with a 9% increased risk of breast cancer, while processed meat consumption shows an 18% increased risk for colorectal cancer and a 6% increased risk for breast cancer (4). However, emerging evidence from burden of proof studies suggests that while there is some evidence of association between unprocessed red meat consumption and breast cancer, the evidence remains weak and insufficient for conclusive recommendations, with 95% uncertainty intervals ranging from 0 to 200 g per day for minimized risk (35). The observed trends across SDI regions and the frontier analysis suggest that the association between red meat consumption and breast cancer may be modulated by socio-economic factors. Previous studies have reported mixed results regarding red meat consumption and breast cancer risk (36, 37). Our findings indicate that the impact of red meat consumption on breast cancer burden may vary depending on the broader dietary and lifestyle context, emphasizing the need for more nuanced dietary recommendations that consider cultural and socio-economic factors (4).

The analysis of health inequalities reveals a nuanced picture of global breast cancer disparities. While absolute inequalities (as measured by the Slope Index) increased for mortality, relative inequalities (measured by the Concentration Index) decreased for both mortality and DALYs. This suggests that while the gap in absolute numbers may be widening, the relative distribution of the disease burden is becoming more equitable. However, the frontier analysis highlights significant disparities, with some countries far exceeding their expected burden based on SDI. These findings underscore the complex interplay between socio-economic development, healthcare access, and cancer outcomes, emphasizing the need for targeted interventions to address these disparities (23, 27, 38).

The use of multiple machine learning algorithms for projecting future breast cancer burden represents a methodological advancement in epidemiological forecasting. The superior performance of ARIMA for male projections and Prophet for female projections highlights the importance of gender-specific modeling approaches. These projections, indicating continued increases in global breast cancer burden through 2050, provide valuable insights for long-term health planning and resource allocation. However, it's crucial to note that these projections assume current trends and do not account for potential breakthroughs in prevention or treatment. Future studies should consider incorporating scenario analyses to account for potential changes in risk factors or treatment efficacy (39–41).

The comparative analysis of eight machine learning models revealed distinct performance patterns across different demographic groups and outcome measures. Our comprehensive evaluation (Supplementary Tables S15, S16) demonstrated that no single model consistently outperformed all others across all scenarios, highlighting the importance of model selection based on specific population characteristics and outcome types. For male breast cancer predictions, ARIMA consistently demonstrated superior accuracy across all metrics, achieving the lowest mean squared error (MSE) values for both deaths (17.35) and DALYs (8,501.30), along with the highest *R*^2^ values (>0.998), indicating excellent model fit. This superior performance may be attributed to ARIMA's ability to capture the more stable, linear trends observed in male breast cancer patterns, which are less influenced by hormonal fluctuations and screening program changes compared to females. Conversely, Prophet emerged as the optimal choice for female breast cancer projections, particularly excelling in handling the complex seasonal patterns and trend changes characteristic of female breast cancer epidemiology. The model's built-in capability to detect and accommodate changepoints in trends proved particularly valuable for capturing the impacts of evolving screening programs, treatment advances, and lifestyle changes on female breast cancer burden. The Prophet model's superior performance in female predictions (*R*^2^ values >0.92 for all measures) reflects its strength in modeling non-linear trends and handling uncertainties in projection scenarios.

Notably, traditional time-series models (TBATS, ETS) and more complex machine learning approaches (ElasticNet, VAR) showed considerably poorer performance, often with negative *R*^2^ values, indicating that model complexity does not necessarily translate to improved prediction accuracy. This finding supports the principle of model parsimony in epidemiological forecasting, where simpler, well-suited models often outperform more complex alternatives. The poor performance of certain models (particularly TBATS and ETS) may be attributed to their tendency to overfit to noise in the data or their inability to appropriately handle the specific characteristics of breast cancer time-series data. These findings have important implications for future epidemiological forecasting studies, suggesting that model selection should be tailored to specific demographic groups and outcome measures rather than applying a one-size-fits-all approach. The demonstrated superiority of ARIMA for males and Prophet for females provides evidence-based guidance for researchers conducting similar burden projections and emphasizes the value of comparative model evaluation in ensuring robust and reliable forecasts.

The findings of this study have significant implications for public health policy and clinical practice. The persistent increase in breast cancer burden, particularly in developing regions, calls for strengthened cancer control programs that encompass primary prevention, early detection, and improved treatment access (42). The observed trends in younger age groups suggest a need to reevaluate screening guidelines and risk assessment tools to better capture early-onset cases (43). Additionally, the gender disparities highlighted in this study underscore the importance of gender-specific approaches in breast cancer awareness and management, particularly for male breast cancer (44).

Current dietary guidelines and policy recommendations emphasize the importance of limiting red meat consumption for cancer prevention. The World Health Organization and various national cancer prevention guidelines recommend reducing red meat intake to decrease cancer risk, with some guidelines suggesting limiting consumption to < 500 g per week (4). Our projections indicating continued increases in breast cancer burden through 2050, particularly in low-SDI regions, underscore the urgent need for targeted dietary interventions. For low-SDI countries, policy recommendations should include: (1) implementing population-level awareness campaigns about the association between high red meat consumption and breast cancer risk; (2) promoting dietary diversification with increased consumption of protective foods such as vegetables, fruits, and legumes, which have been shown to reduce breast cancer risk by 3–4% per 100 g daily intake (5); (3) developing culturally appropriate dietary guidelines that consider local food systems and economic constraints; and (4) integrating nutrition education into existing healthcare infrastructure and cancer prevention programs. The economic burden of breast cancer, combined with the preventable nature of diet-related risk, makes these interventions particularly cost-effective for resource-limited settings.

## Limitation

While this study provides comprehensive insights into global breast cancer trends, several limitations should be acknowledged. The ecological nature of the study precludes causal inferences about the relationship between red meat consumption and breast cancer burden. Future research should incorporate individual-level data to better elucidate this relationship. Additionally, the projections, while robust, are based on historical trends and may not fully capture future changes in risk factors or treatment advancements. Further studies should aim to incorporate more detailed risk factor data and explore the potential impact of emerging prevention and treatment strategies on future breast cancer burden (45, 46). Additionally, the Global Burden of Disease study methodology, while robust and comprehensive, introduces inherent uncertainties in disease burden estimates. These uncertainties arise from variations in data quality and availability across different countries and regions, differences in case definitions and diagnostic practices, and the use of statistical modeling to estimate burden in locations with limited data. The comparative risk assessment framework used to attribute breast cancer burden to high red meat consumption relies on meta-analyses of observational studies, which may be subject to confounding factors and measurement errors. Furthermore, the disability weights used in DALY calculations are based on population surveys that may not fully capture the lived experience of breast cancer patients across different cultural contexts (6, 10). Future studies should incorporate sensitivity analyses to better quantify these uncertainties and their impact on burden estimates and projections.

## Conclusions

This comprehensive analysis of breast cancer burden attributable to high red meat consumption from 1990 to 2021 reveals significant global disparities and concerning future trends that demand urgent public health attention. While age-standardized mortality and DALY rates have declined in high-SDI regions, the absolute burden continues to rise globally, with particularly pronounced increases in low- and middle-SDI countries and among younger women aged 25–34 years. Our machine learning projections indicate that by 2050, the global burden will reach 99,749 deaths and 2,965,835 DALYs among females, and 1,982 deaths and 55,585 DALYs among males, representing substantial increases from current levels. The persistent health inequalities demonstrated through our slope index and concentration index analyses, combined with the frontier analysis revealing significant disparities in disease control effectiveness across countries, underscore the complex interplay between dietary risk factors, socioeconomic development, and healthcare access. These findings highlight the critical need for targeted, culturally appropriate dietary interventions and policy frameworks that address modifiable risk factors while considering regional socioeconomic constraints, particularly in lower-SDI regions where the burden is projected to increase most dramatically, thereby providing essential evidence for informed public health planning and resource allocation strategies to mitigate the growing global impact of diet-related breast cancer burden.

## Data Availability

The original contributions presented in the study are included in the article/Supplementary material, further inquiries can be directed to the corresponding author.

## Supplementary material

Supplementary Figure S1 Decomposition Analysis of Changes in Breast Cancer Burden Attributable to High Red Meat Consumption, 1990–2021. **(A)** Decomposition of changes in mortality, both sexes. **(B)** Decomposition of changes in mortality, males. **(C)** Decomposition of changes in mortality, females. **(D)** Decomposition of changes in DALYs, both sexes. **(E)** Decomposition of changes in DALYs, males. **(F)** Decomposition of changes in DALYs, females.

Supplementary Figure S2 Correlation Between SDI and Age-Standardized Rates of Breast Cancer Burden Attributable to High Red Meat Consumption Across 204 Countries. (A) SDI vs. age-standardized mortality rates, 1990. (B) SDI vs. age-standardized DALY rates, 1990.

Supplementary Figure S3 Correlation Between SDI and Age-Standardized Rates of Breast Cancer Burden Attributable to High Red Meat Consumption Across GBD Regions, 2021. **(A)** SDI vs. age-standardized mortality rates for global and 21 GBD regions. **(B)** SDI vs. age-standardized DALY rates for global and 21 GBD regions.

Supplementary Figure S4 Frontier Analysis of Breast Cancer Burden Attributable to High Red Meat Consumption in Relation to SDI. **(A)** Frontier analysis of age-standardized mortality rates across 204 countries, 1990–2021. **(B)** Frontier analysis of age-standardized mortality rates across 204 countries, 2021. **(C)** Frontier analysis of age-standardized DALY rates across 204 countries, 1990–2021. **(D)** Frontier analysis of age-standardized DALY rates across 204 countries, 2021.

Supplementary Table S1 Number, age-standardized rate and estimated annual percentage change of breast cancer mortality attributable to high red meat intake globally and by sex in population aged 25 and above, 1990–2021.

Supplementary Table S2 Number, age-standardized rate and estimated annual percentage change of breast cancer DALYs attributable to high red meat intake globally and by sex in population aged 25 and above, 1990–2021.

Supplementary Table S3 Age-Specific Breast Cancer Mortality Rates and Trends Attributable to High Red Meat Intake by Sex, 1990–2021.

Supplementary Table S4 Age-Specific Breast Cancer Disability-Adjusted Life Years (DALYs) and Trends Attributable to High Red Meat Intake by Sex, 1990–2021.

Supplementary Table S5 Trends in Age-Standardized Breast Cancer Mortality Rates Attributable to High Red Meat Intake Across 204 Countries, 1990–2021.

Supplementary Table S6 Trends in Age-Standardized Breast Cancer Disability-Adjusted Life Years (DALYs) Attributable to High Red Meat Intake Across 204 Countries, 1990–2021.

Supplementary Table S7 Age-Period-Cohort Analysis of Breast Cancer Mortality and DALYs Attributable to High Red Meat Intake (Overall Population), 1990–2021.

Supplementary Table S8 Age-Period-Cohort Analysis of Breast Cancer Mortality and DALYs Attributable to High Red Meat Intake (Males), 1990–2021.

Supplementary Table S9 Age-Period-Cohort Analysis of Breast Cancer Mortality and DALYs Attributable to High Red Meat Intake (Females), 1990–2021.

Supplementary Table S10 Decomposition Analysis of Changes in Breast Cancer Mortality Attributable to High Red Meat Intake by Location, SDI, and Gender, 1990–2021.

Supplementary Table S11 Decomposition Analysis of Changes in Breast Cancer Disability-Adjusted Life Years (DALYs) Attributable to High Red Meat Intake by Location, SDI, and Gender, 1990–2021.

Supplementary Table S12 Trends in slope index of inequality and concentration index for breast cancer mortality and DALYs attributable to high red meat intake, global population aged 25 and above, 1990–2021.

Supplementary Table S13 Frontier analysis of breast cancer mortality rates attributable to high red meat intake among aged 25 and above in 204 countries and territories worldwide.

Supplementary Table S14 Frontier analysis of age-standardized DALY rates due to breast cancer attributable to high red meat intake among aged 25 and above in 204 countries and territories worldwide.

Supplementary Table S15 Machine Learning Algorithms Comprehensive Comparison Table.

Supplementary Table S16 Performance Metrics Comparison of Eight Machine Learning Models for Breast Cancer Burden Prediction Attributable to High Red Meat Intake by Sex and Measure Type.
