# Supplementary figures and images for: Global trends and health workforce analysis of breast cancer burden from high red meat consumption 1990–2050 using machine learning approach
Source: Front Nutr. 2025 Aug 14;12:1576043. doi: 10.3389/fnut.2025.1576043 (PMC12390816; doi:10.3389/fnut.2025.1576043)

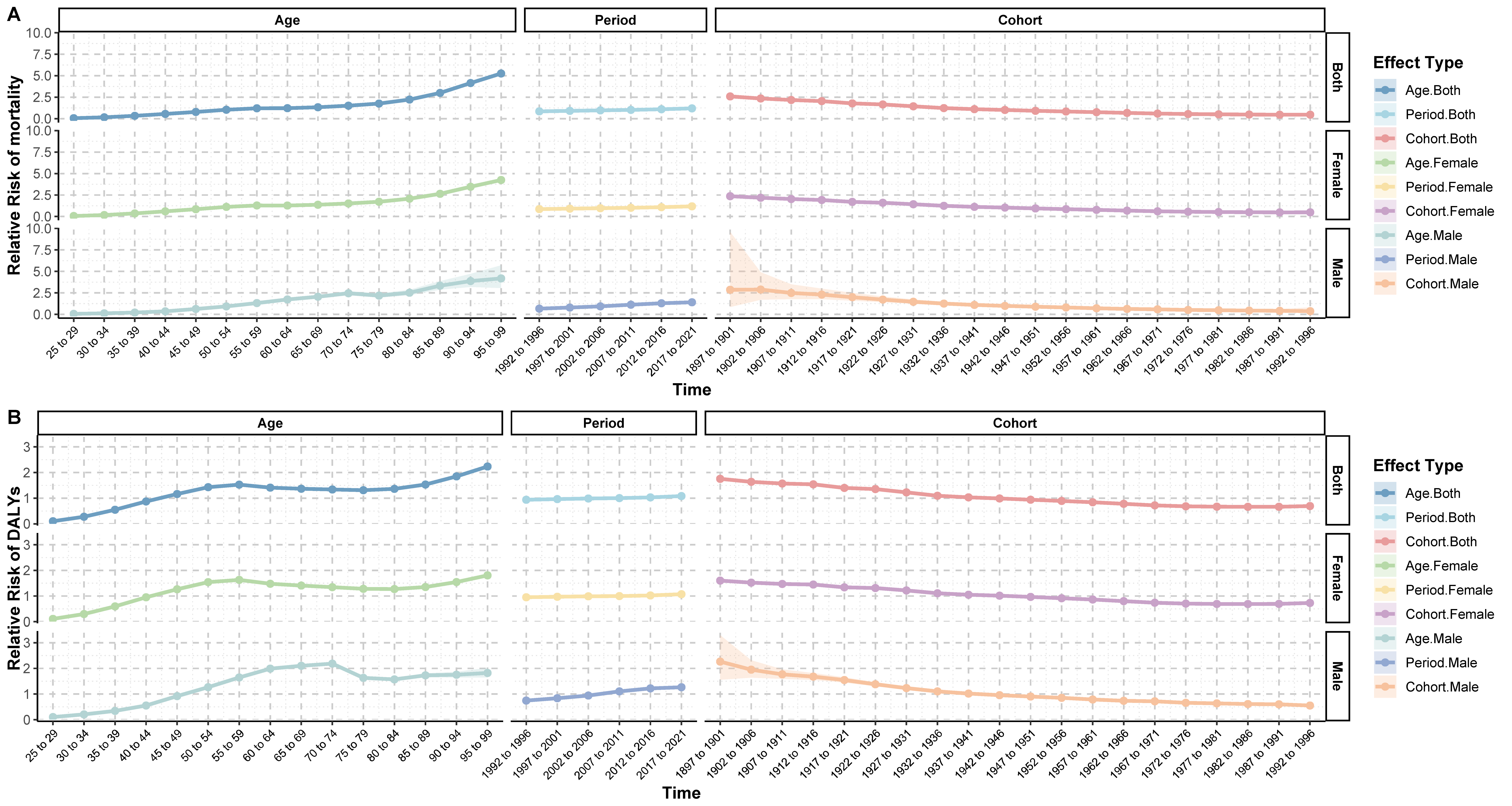

Supplement: Supplementary Figure S1 — Decomposition Analysis of Changes in Breast Cancer Burden Attributable to High Red Meat Consumption, 1990–2021. (A) Decomposition of changes in mortality, both sexes. (B) Decomposition of changes in mortality, males. (C) Decomposition of changes in mortality, females. (D) Decomposition of changes in DALYs, both sexes. (E) Decomposition of changes in DALYs, males. (F) Decomposition of changes in DALYs, females. [file Image_1.TIFF]

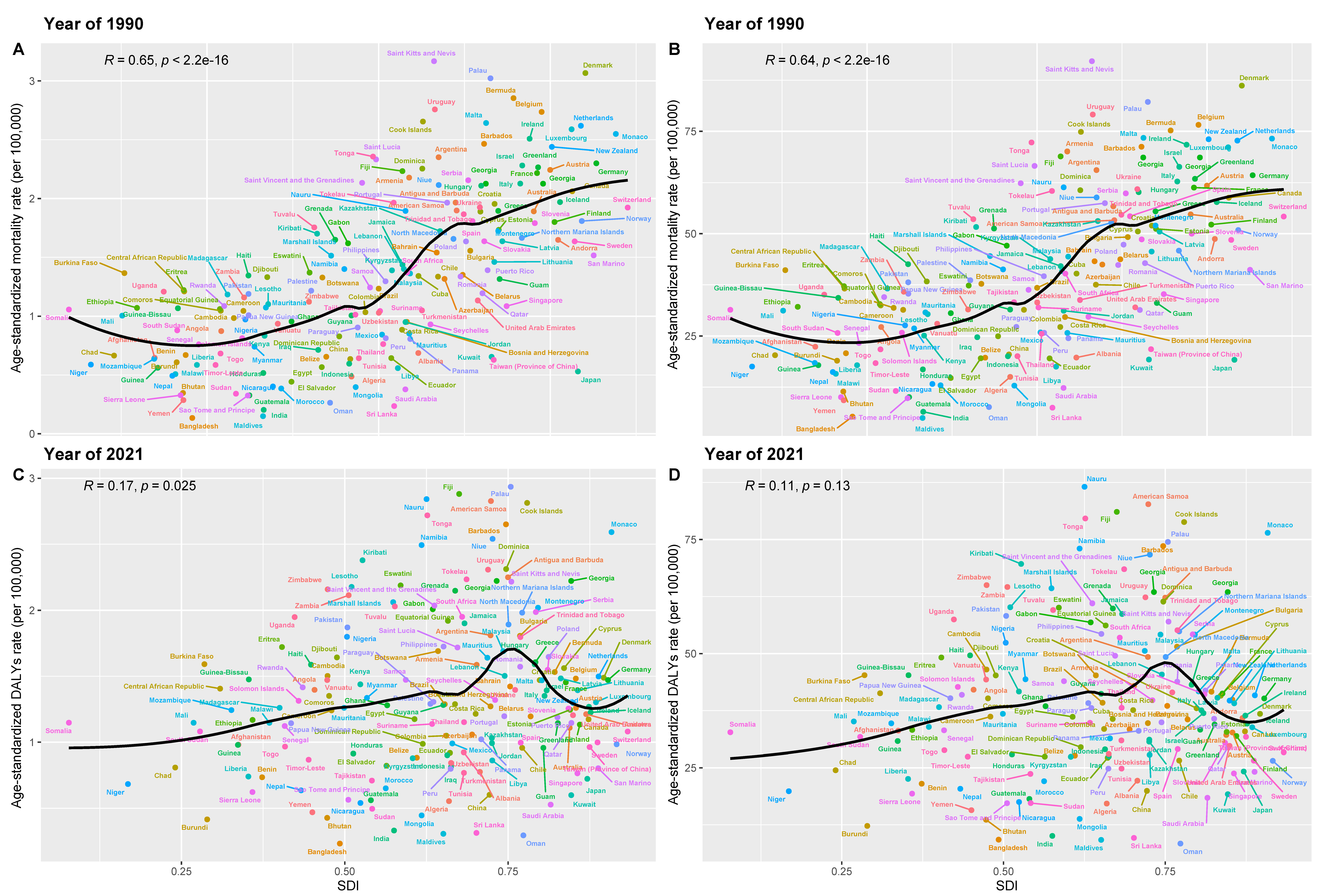

Supplement: Supplementary Figure S2 — Correlation Between SDI and Age-Standardized Rates of Breast Cancer Burden Attributable to High Red Meat Consumption Across 204 Countries. (A) SDI vs. age-standardized mortality rates, 1990. (B) SDI vs. age-standardized DALY rates, 1990. [file Image_2.TIFF]

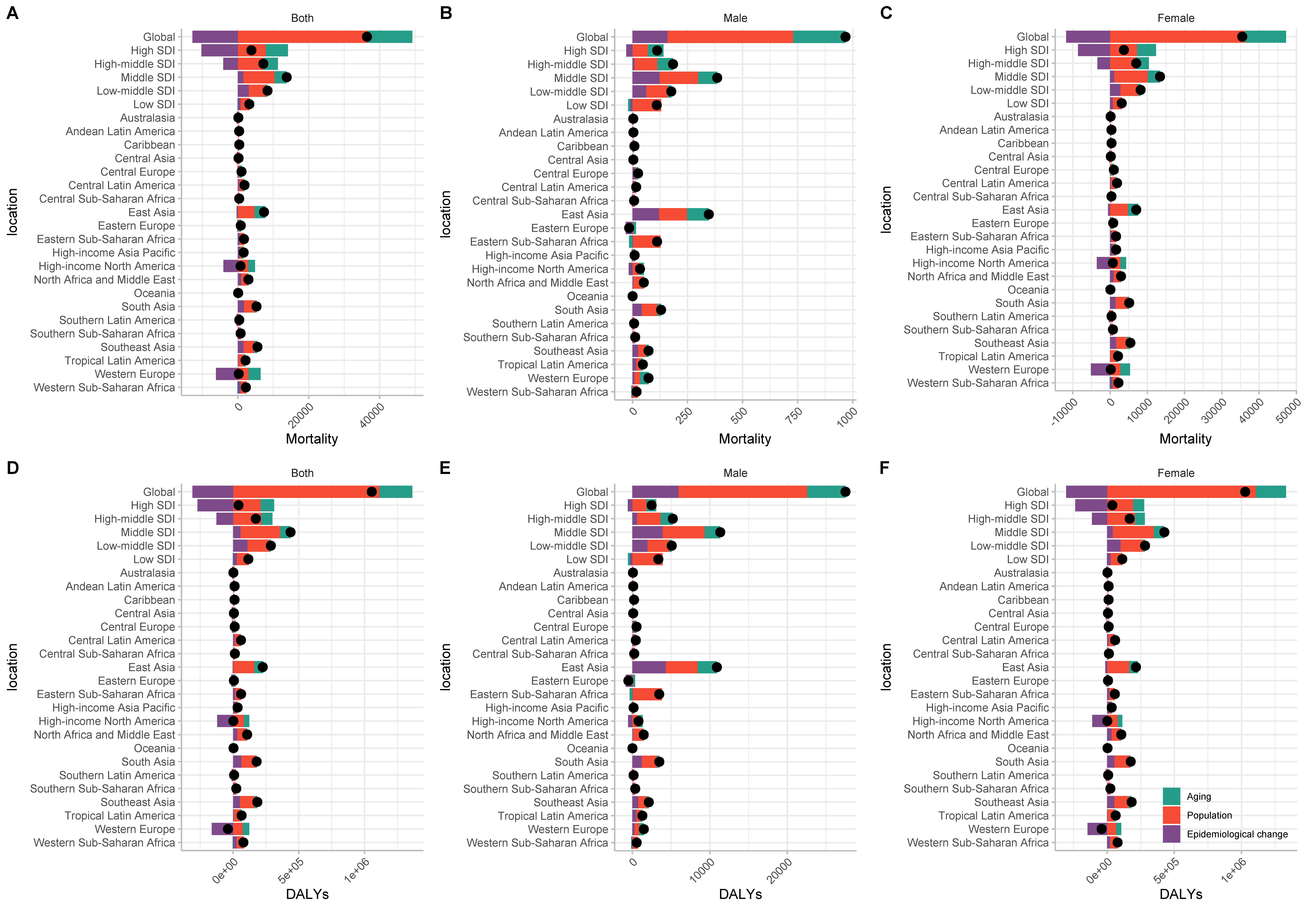

Supplement: Supplementary Figure S3 — Correlation Between SDI and Age-Standardized Rates of Breast Cancer Burden Attributable to High Red Meat Consumption Across GBD Regions, 2021. (A) SDI vs. age-standardized mortality rates for global and 21 GBD regions. (B) SDI vs. age-standardized DALY rates for global and 21 GBD regions. [file Image_3.TIFF]
